# METTL3 inhibition attenuates AFB_1_-induced hepatic fibrosis by suppressing m^6^A-mediated hepatic stellate cell activation

**DOI:** 10.1186/s40104-026-01380-4

**Published:** 2026-05-28

**Authors:** Yulan Zhao, Yue Feng, Wenbo Yuan, Tingyu Zhang, Ruqian Zhao

**Affiliations:** 1 MOE Joint International Research Laboratory of Animal Health & Food Safety, Nanjing Agricultural University, Nanjing, Jiangsu People’s Republic of China; 2 Key Laboratory of Animal Physiology & Biochemistry, College of Veterinary Medicine, Nanjing Agricultural University, Nanjing, Jiangsu People’s Republic of China; 3 College of Animal Sciences, Fujian Agriculture and Forestry University, Fuzhou, Fujian People’s Republic of China

**Keywords:** Aflatoxin B_1_, Extracellular matrix, Hepatic stellate cells, Liver fibrosis, M^6^A modification

## Abstract

**Background:**

Aflatoxin B_1_ (AFB_1_) is a potent hepatotoxic mycotoxin and a major environmental risk factor for hepatocellular carcinoma (HCC). Hepatic fibrosis is a critical intermediate stage in this process, and METTL3-mediated m^6^A modification may represent an important post-transcriptional mechanism linking AFB_1_-induced liver injury to fibrogenic progression.

**Methods:**

AFB_1_-induced hepatic fibrosis was evaluated using in vivo mouse models and in vitro cultured hepatic stellate cells (HSC). Global m^6^A methylation and methyltransferase-like 3 (METTL3) expression were assessed by liquid chromatography-mass spectrometry, Western blotting, single-nucleus RNA sequencing, and quantitative real-time PCR. METTL3 was inhibited using small interfering RNA or the selective inhibitor STM2457. Molecular docking was performed to identify potential METTL3-binding compounds, followed by functional validation.

**Results:**

AFB_1_ exposure promoted hepatic fibrosis and HSC activation, accompanied by global m^6^A hypermethylation and upregulation of METTL3. METTL3 increased the m^6^A modification of collagen-related transcripts, enhancing their stability and promoting extracellular matrix production in a *YTHDF1*-dependent manner. Inhibition of METTL3 suppressed HSC activation and fibrotic gene expression both in vitro and in vivo. Molecular docking identified saxagliptin as a potential METTL3-binding compound, which reduced AFB_1_-induced HSC activation and extracellular matrix accumulation, consistent with the effects of STM2457.

**Conclusions:**

These findings indicate that METTL3 functions as a post-transcriptional regulator in AFB_1_-induced liver fibrosis via m^6^A modification. METTL3 inhibition, achieved via genetic knockdown or selective inhibitors, affects HSC activation and fibrotic gene expression, supporting its role as a therapeutic target in AFB_1_-induced liver fibrosis.

**Supplementary Information:**

The online version contains supplementary material available at 10.1186/s40104-026-01380-4.

## Introduction

Liver fibrosis, the accumulation of scar tissue resulting from chronic liver injury, is a critical pathological process driving the progression towards end-stage liver diseases, including cirrhosis and hepatocellular carcinoma (HCC) [1, 2]. This scarring arises from liver fibrosis, the dynamic and active process involving activation of hepatic stellate cells (HSC), excessive extracellular matrix deposition, and tissue remodeling. Liver injury initiating this cascade can stem from various exogenous toxins, pathogenic infections, and endogenous metabolic disorders, such as dysregulated lipid metabolism, mycotoxin exposure, and parasitic infections [3–5]. Aflatoxin B_1_ (AFB_1_) is regarded as the most toxic mycotoxin, capable of inducing significant structural and functional damage to the liver and other organs in humans, experimental animals, and livestock [6]. Given its high toxicity, widespread exposure to AFB_1_ represents a substantial public health and agricultural concern. Indeed, previous surveys have reported that the contamination rate of AFB_1_ in Chinese feed ranges from 20.0% to 100%, with average concentrations spanning 1.2 to 728.7 μg/kg. At the molecular level, the hepatotoxic and carcinogenic effects of AFB_1_ are primarily attributed to its bioactivation, whereby AFB_1_ is metabolized into the highly reactive aflatoxin B_1_-8,9-epoxide (AFBO), which forms covalent DNA adducts, leading to genetic mutations and ultimately driving hepatocarcinogenesis [7, 8]. It is estimated that 4.5 billion people worldwide are exposed to AFB_1_ and may develop HCC, and about 5%−28% of global HCC cases are attributable to aflatoxin exposure [9]. Given AFB_1_’s potency as a hepatotoxin and major carcinogen, and considering that liver fibrosis represents the critical, active intermediate stage bridging liver injury and HCC, further exploration of the mechanisms underlying AFB_1_-induced liver fibrosis, particularly its cell-specific drivers and the relatively understudied early initiation phase, is warranted.

Hepatic stellate cells are the primary mediators of liver fibrosis, becoming activated in response to liver injury and contributing to excessive extracellular matrix (ECM) deposition [10]. The activation of HSC is driven by multiple mechanisms, including damage-associated molecular patterns (DAMPs) released by injured hepatocytes and pro-fibrotic factors secreted by Kupffer cells [11]. In AFB_1_-induced hepatotoxicity models, research has predominantly focused on the detrimental effects of AFB_1_ on hepatocytes, such as oxidative stress, lipid accumulation, and pyroptosis [12]. Notably, AFB_1_ exposure has been shown to activate the p53-Parkin-dependent mitophagy pathway in hepatocytes, leading to the release of CD63-enriched extracellular vesicles (EVs), which in turn promote HSC activation and accelerate liver fibrosis progression [13]. However, whether AFB_1_ can directly activate HSC and the precise mechanisms underlying this process remain unexplored.

N^6^-methyladenosine (m^6^A) is the most prevalent RNA modification in the eukaryotic transcriptome, dynamically regulating RNA metabolism, including nuclear export, splicing, and stability [14]. Accumulating evidence has linked m^6^A modifications to the pathogenesis of liver diseases such as nonalcoholic fatty liver disease (NAFLD) [15], hepatitis and HCC [16, 17]. m^6^A modification also plays a crucial role in fibrosis. For instance, the m^6^A reader IGF2BP2 regulates glycolytic metabolism and mediates histone lactylation, thereby promoting HSC activation and exacerbating liver fibrosis [18]. Additionally, m^6^A modification has been shown to enhance ferroptosis in HSC through the autophagy signaling pathway, further contributing to fibrosis progression [19]. Given the well-established involvement of m^6^A in liver fibrosis, it remains unclear whether m^6^A modification also plays a regulatory role in AFB_1_-induced fibrosis and whether targeting m^6^A-related pathways could serve as a therapeutic approach for AFB_1_-induced liver injury. As the main m^6^A methyltransferase, METTL3 may mediate AFB_1_-driven HSC activation and fibrogenesis, making it a critical target to explore in this context.

In this study, we identified m^6^A methyltransferase METTL3 as a crucial regulator of AFB_1_-induced liver fibrosis and HSC activation. Furthermore, we demonstrated that saxagliptin effectively attenuates AFB_1_-induced liver fibrosis by targeting METTL3, highlighting a novel therapeutic strategy for mitigating AFB_1_-related liver damage. Our findings provide new insights into the epitranscriptional regulation of AFB_1_-induced fibrosis and suggest that targeting m^6^A methylation may represent a potential strategy for treating AFB_1_-associated liver diseases.

## Methods

### Animals

Eight-week-old male SPF-grade C57BL/6 J mice were housed with ad libitum access to standard chow and purified water. After a one-week acclimatization, they were randomly assigned to experimental groups (*n* = 12 per group). The control group (CON) received 0.2 mL olive oil by gavage daily, while the AFB_1_ group was administered 0.45 mg/kg AFB_1_ in 0.2 mL olive oil. Additional groups included AFB_1_ + STM2457, receiving intraperitoneal injections of 20 mg/kg STM2457 ethanol solution, and AFB_1_ + Sax, receiving 0.2 mL of 20 mg/kg saxagliptin aqueous solution by gavage. The experiment lasted 21 d.

Before sampling, mice were fasted for 6 h and anesthetized with 0.2 mL of 25% urethane via intraperitoneal injection. Blood was collected via orbital enucleation, left overnight at 4 °C, centrifuged (4 °C, 2,500 × *g*, 15 min), and the serum stored at −20 °C. Mice were euthanized by cervical dislocation, and liver tissues were harvested and stored at −80 °C.

### Isolation of primary HSC

Anesthetized mice were perfused with calcium-free Hanks’ balanced salt solution, followed by collagenase digestion. Hepatocytes were removed through mechanical dissociation, filtration with calcium-containing Hanks’ balanced salt solution, and centrifugation at 3,500 r/min for 5 min. The supernatant, containing nonparenchymal cells, was layered onto a Percoll gradient (25% and 50%; Solarbio, Beijing, China) and centrifuged at 2,000 × *g* for 10 min at 4 °C. The interphase ring, enriched with HSC, was collected and washed twice before RNA and protein extraction.

### Cell culture and treatment

The murine SV40-immortalized hepatic stellate cell line JS1 (BNCC359737, BeNa Culture Collection, Beijing, China) was maintained in DMEM (319-005, Wisent, China) supplemented with 10% fetal bovine serum (10099141, Gibco, USA) and 1% penicillin/streptomycin (15070063, Gibco, USA) at 37 °C with 5% CO_2_. Once cells reached 80% confluence, they were exposed to 20 μmol/L AFB_1_ (MSS1003, Pribolab, Singapore) for 12 h.

### Histopathology and immunofluorescence

Fresh liver tissue was fixed in 4% paraformaldehyde, embedded in paraffin, and sectioned at 4 μm. Liver sections were stained with hematoxylin and eosin, Sirius Red, and Masson’s trichrome. Immunofluorescence staining was performed to detect α-SMA (14395-1-AP, Proteintech, China, 1:400), Collagen I (BA0325, Boster Biological Technology, China, 1:100), and METTL3 (AB98009, Abcam, USA, 1:500). DAPI (Molecular Probes, USA) was used to stain cell nuclei.

### Cytokine quantification

The concentrations of IL-1β, IL-6, and TGF-β1 in mouse serum were quantified using commercially available enzyme-linked immunosorbent assay (ELISA) kits (Multisciences, Hangzhou, China) according to the manufacturer’s instructions. All assays rely on specific antigen–antibody recognition to ensure sensitive and accurate detection, and measurements were performed in duplicate.

### Single nuclei RNA sequencing (SnRNA-seq) and data analysis

Liver nuclei from two mice in each group were pooled and loaded into 10 × Genomics Chromium Single Nuclei chips. Library construction was performed using the Chromium Single Cell 3′ GEM Library & Gel Bead Kit v3 (10 × Genomics, cat. no. PN-CA). Sequencing data were analyzed using a modified Seurat (v3) single-cell analysis workflow. Slingshot was applied to infer pseudo-time trajectories.

### Transcriptome sequencing (RNA-seq)

Total RNA was extracted using TRIzol Reagent (TSP401, Tsingke Biotech, China) following the manufacturer’s protocol. RNA purity and quality were assessed accordingly. Library construction and RNA sequencing were performed by OmicShare Co., Ltd. (Beijing, China).

### RNA isolation and real-time quantitative PCR (qPCR)

Total RNA was extracted using TRIzol Reagent (TSP401, Tsingke Biotech, China), and 1 μg of RNA was reverse-transcribed into cDNA using a HiScript® II Reverse Transcriptase kit (R233-01, Vazyme, China) following the manufacturer's instructions. For qPCR, 2 μL of diluted cDNA (1:20, v/v) was used with a QuantStudio™ 6 Flex Real-Time PCR System (384-well, Thermo Scientific, USA). Primers were synthesized by Tsingke Biotech (Nanjing, China) (Table S1). *GAPDH*, an unaffected gene, was used as the reference. Data were analyzed using the 2^−ΔΔCT^ method.

### Protein extraction and Western blot analysis

Protein concentrations were determined using the BCA Protein Assay kit (DQ111-01, TransGen Biotech, China). A total of 20 μg of protein was loaded onto 10% or 15% SDS-PAGE gels, separated by electrophoresis, and transferred to a nitrocellulose membrane. Primary antibodies used for Western blot are listed in Table S2, with α-tubulin as the internal control. Protein bands were visualized using the VersaDoc 4000MP system (Bio-Rad, USA), and band intensity was analyzed using Quantity One software (Bio-Rad, USA).

### Methylated RNA immunoprecipitation (MeRIP) assay

Total RNA was extracted and fragmented chemically at 94 °C for 5 min in a buffer (0.1 mol/L ZnCl_2_, 0.1 mol/L Tris-HCl, pH 7.0) to 150–300 bp, then ethanol-precipitated and purified using an EasyPure RNA Purification Kit (ER701-01; TransGen, China). Fragmented RNA (40 μg) was precleared with protein A/G agarose beads (40 μL, sc-2003, Santa Cruz Biotechnology, USA) and 40U RNase inhibitor overnight at 4 °C. The mixture was incubated with 2 μg m^6^A antibody (ab151230, Abcam, USA) overnight at 4 °C, with a negative control using normal IgG (2729S, Cell Signaling Technology, USA). Protein A/G agarose beads were used to capture the immunoprecipitated complexes. RNA was eluted with 300 μL elution buffer (5 mmol/L Tris-HCl, 1 mmol/L EDTA, 0.05% sodium dodecyl sulfate) and 20 μg proteinase K for 1 h at 60 °C. After phenol extraction and ethanol precipitation, the input and m^6^A-enriched RNA were reverse transcribed with random hexamers. m^6^A enrichment in specific transcripts was analyzed by qPCR, using primers listed in Table S3.

### SELECT for m^6^A detection

m^6^A peak-containing sequences of collagen mRNAs were analyzed for specific m^6^A site prediction using SRAMP (http://www.cuilab.cn/sramp). High or very high-confidence m^6^A site was chosen for each mRNA and validated using the single-base elongation- and ligation-based qPCR method ("SELECT") [20]. The SELECT products at the target sites were normalized to the corresponding RNA input levels. Primer sequences used in the SELECT assay are provided in Table S4.

### siRNA transfection and inhibitor treatment

JS1 cells at 60% confluence were transfected with siRNA targeting *METTL3*. Transfection was performed using Lipofectamine 2000 (Invitrogen, USA) according to the manufacturer’s protocol.

### RNA decay assay

JS1 cells were seeded in 6-well plates and treated with or without *TGF-β1* or *YTHDF1* siRNA. Actinomycin D (HY-17559, MCE, USA) was then added to a final concentration of 5 μg/mL to block transcription. Cells were harvested at 0-, 3- and 6-h post-treatment, and total RNA was extracted for RT-PCR to assess the relative collagen mRNA levels (normalized to 0 h).

### Molecular docking

The tertiary structure of METTL3 was retrieved from the Protein Data Bank (PDB) and preprocessed by removing water molecules and other non-essential components. The chemical structures of STM2457 and saxagliptin were optimized using Chem3D. Molecular docking was performed using AutoDock Vina, where METTL3, STM2457, and saxagliptin were converted into docking-compatible formats, and the search space and binding site parameters were defined. The docking simulation explored multiple binding modes through an algorithm-based approach, and the binding affinities of different conformations were calculated. The optimal METTL3-STM2457 and METTL3-saxagliptin binding conformations were selected based on binding energy and interaction analysis. Docking results were visualized using software such as PyMOL.

### Statistical analysis

Data are expressed as mean ± SE from at least two independent experiments. Group differences were analyzed using Student’s *t*-test or two-way ANOVA followed by Tukey’s post-hoc test for multiple comparisons (SPSS 20.0, IBM, Armonk, NY, USA). Statistical significance was set at ^*^P < 0.05 or ^**^P < 0.01.

## Results

### Hepatic fibrosis caused by AFB_1_ is associated with global RNA m^6^A hypermethylation

**Fig. 1:**
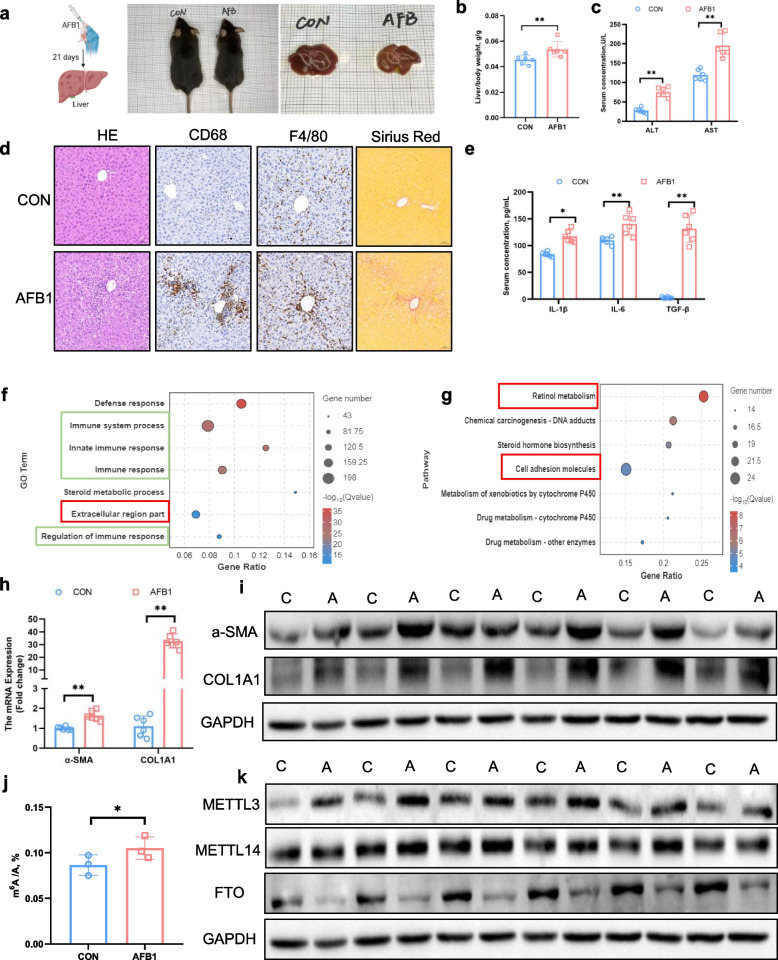
AFB1-associated hepatic fibrosis is accompanied by global RNA m^6^A hypermethylation. **a** Schematic representation of the mouse model, along with the body weight and liver morphology. **b** Ratio of liver weight to body weight (*n* = 6). **c** Plasma AST and ALT activities (*n* = 6). **d** HE, CD68, F4/80 and Sirius Red staining of liver sections (*n* = 3). **e** Hepatic concentrations of inflammatory cytokines (*n* = 6). **f** Gene Ontology (GO) enrichment analysis of differentially expressed genes (DEGs) in the liver (*n* = 3). **g** Kyoto Encyclopedia of Genes and Genomes (KEGG) enrichment analysis of DEGs in the liver (*n* = 3). **h **and** i** Hepatic expression of *α-SMA* and *COL1A1* mRNA and protein (*n* = 6). **j** Hepatic global m^6^A level detected by high-performance liquid chromatography with tandem mass spectrometry (*n* = 3). **k** Hepatic protein expression of METTL3, METTL14 and FTO (*n* = 6). Data are presented as mean ± SEM; ^*^P < 0.05, ^**^P < 0.01

After administering AFB_1_ to mice via gavage for 21 d, we collected liver tissues for subsequent analyses (Fig. 1a). AFB_1_ exposure resulted in a significant decrease (*P* < 0.01) in both food intake and body weight (Fig. S1a and b). Additionally, AFB_1_ caused a marked increase in the liver-to-body weight ratio (*P* < 0.01), along with elevated serum levels of AST and ALT (*P* < 0.01; Fig. 1b and c). Histopathological examination of the liver revealed evident inflammatory infiltration and excessive collagen deposition in the AFB_1_-treated mice liver (Fig. 1d). Furthermore, AFB_1_ treatment significantly upregulated serum levels of inflammatory cytokines, including IL-1β, IL-6, and TGF-β (*P* < 0.05; Fig. 1e). RNA sequencing of liver tissues from control and AFB_1_-treated mice demonstrated distinct clustering of RNA profiles between the two groups as shown by PCA analysis (Fig. S1c). Volcano plots of differential expression analysis revealed that AFB_1_ exposure upregulated 864 genes and downregulated 1,287 genes (Fig. S1d). GO and KEGG pathway enrichment analyses highlighted significant activation of inflammation-related pathways, including immune response and extracellular region part (Fig. 1f and g). These findings indicate that AFB_1_-induced liver damage in mice is associated with inflammation and fibrosis. As fibrosis is a hallmark of liver injury repair, we assessed the expression of fibrosis markers and found significant upregulation of α-SMA and COL1A1 at both the gene and protein levels (*P* < 0.01; Fig. 1h, i and Fig. S1e). Furthermore, m^6^A mass spectrometry analysis of liver RNA revealed a significant increase in m^6^A modifications following AFB_1_ treatment (*P* < 0.01; Fig. 1j), accompanied by a notable upregulation of the methyltransferase METTL3 at both gene and protein levels, and a downregulation of FTO at the protein level (*P* < 0.05; Fig. 1k and Fig. S1f, g).

### snRNA-seq reveals AFB_1_-induced m^6^A enzyme redistribution in HSC

**Fig. 2:**
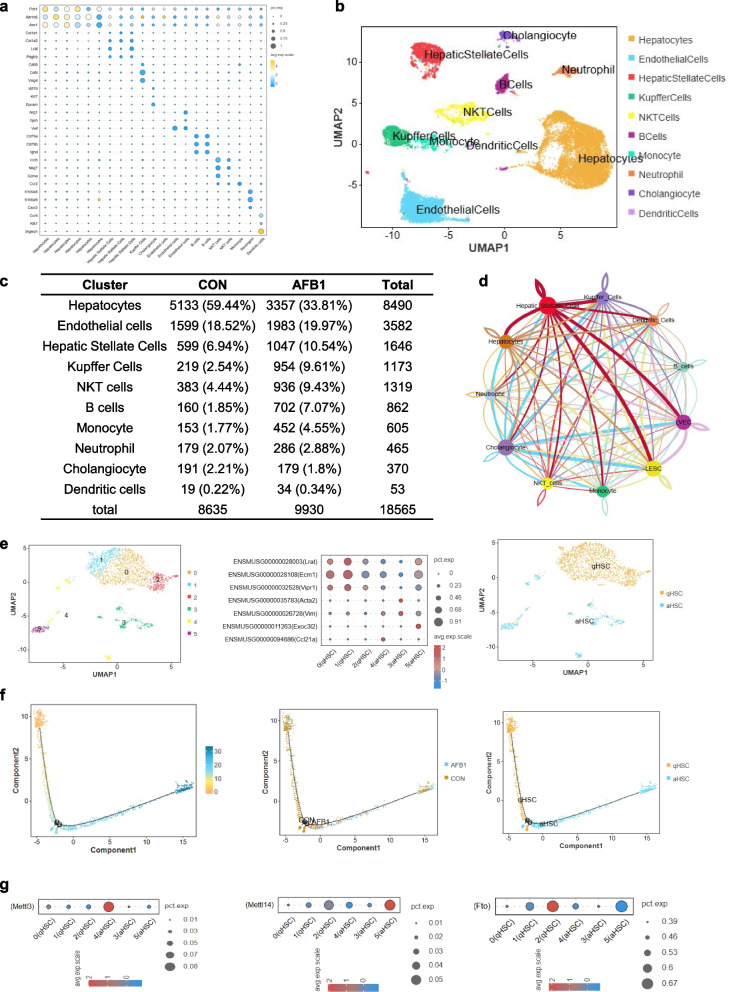
snRNA-seq reveals distribution of m^6^A-metabolizing enzymes in HSC. **a** Dot plot showing gene expression patterns across different liver cell types. **b** UMAP visualization depicting the spatial distribution of all liver cell types. **c** Proportions of different cell types in the liver. **d** Cellular communication analysis. **e** UMAP visualization identifying subpopulations of qHSC and aHSC within HSC. **f** Proposed time-series analysis of HSC dynamics. **g** Expression levels of *METTL3*, *METTL14* and *FTO* transcripts in qHSC and aHSC

We performed snRNA-seq library construction on liver tissues from both CON and AFB_1_ mice. After filtering, a total of 18,560 nuclei were obtained, with 9,930 from the control group and 8,653 from the AFB_1_ group (Fig. S2a). UMAP dimensionality reduction was used for visualization, and cells with similar genomic expression profiles were clustered into 21 subpopulations (Fig. S2b). Cell type-specific marker gene expression was then used to annotate these subpopulations (Fig. 2a). The results showed that the snRNA-seq captured the main hepatic cell types, including hepatocytes, endothelial cells, hepatic stellate cells, Kupffer cells, NKT cells, B cells, monocyte, neutrophil, cholangiocyte and dendritic cells (Fig. 2b). The expression of characteristic genes for different cell types is shown in Fig. S2c. After clustering, the percentage of each cell type was quantified. AFB_1_ treatment led to a reduction in the proportion of hepatocytes, with an increase in the proportion of other cell types, particularly endothelial cells, hepatic stellate cells, Kupffer cells, and NKT cells (Fig. 2c). Cell–cell communication analysis of the clustered cells revealed that HSC had the strongest communication signal (Fig. 2d).

To further investigate the status of HSC, we re-clustered the stellate cell subpopulation and annotated them based on the marker genes of quiescent (qHSC) and activated (aHSC) hepatic stellate cells (Fig. 2e). This analysis revealed that AFB_1_ treatment induces the activation of HSC. Further, pseudotime analysis of the clustered HSC showed a distinct transition point, which marked the boundary between the CON and AFB_1_ groups as well as between qHSC and aHSC, providing additional evidence that AFB_1_ promotes stellate cell activation. Moreover, we observed that the m^6^A methyltransferase *METTL3* exhibited the highest abundance in aHSC, while the demethylase *FTO* was most abundant in qHSC (Fig. 2f), suggesting that m^6^A dynamics play a crucial role in the activation of HSC.

### AFB_1_ activates HSC accompanied by increased m^6^A modification

**Fig. 3:**
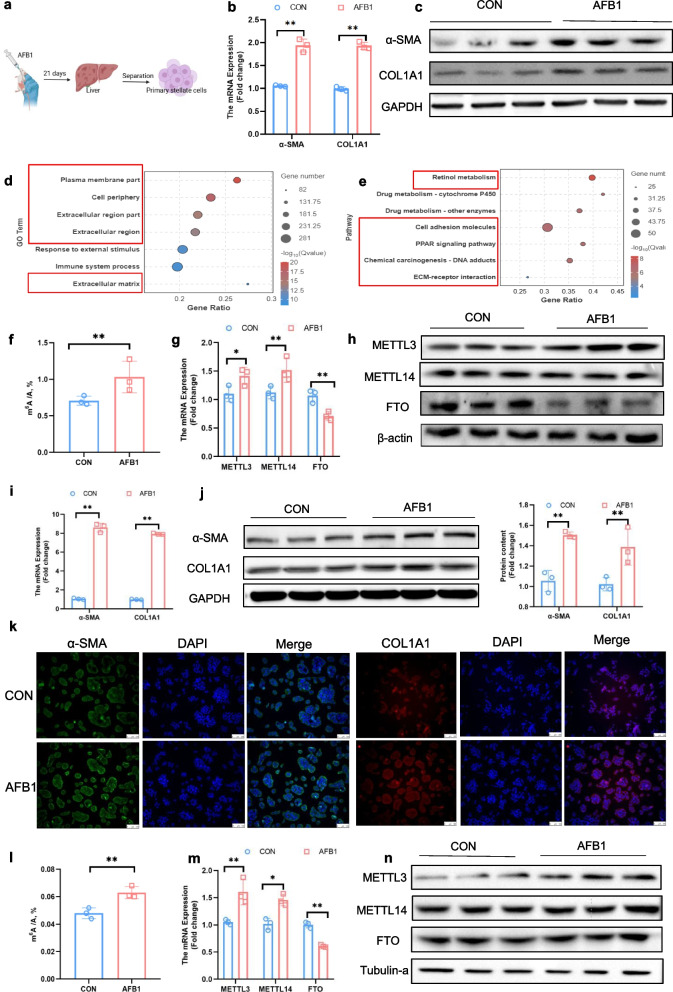
AFB_1_-induced HSC activation is accompanied by increased m^6^A modification. **a** Schematic illustration of the isolation and processing of primary HSC. **b **and** c** mRNA and protein expression levels of α-SMA and COL1A1 in primary HSC (*n* = 3). **d** GO enrichment analysis of DEGs in primary HSC (*n* = 3). **e** KEGG enrichment analysis of DEGs in primary HSC (*n* = 3). **f** Global m^6^A levels in primary HSC detected by high-performance liquid chromatography-tandem mass spectrometry (*n* = 3). **g **and** h** mRNA and protein expression levels of METTL3, METTL14, and FTO in primary HSC (*n* = 3). **i **and** j** mRNA and protein expression levels of α-SMA and COL1A1 in JS-1 cells (*n* = 3). **k** Immunofluorescent staining of α-SMA and COL1A1 proteins. Nucleus was stained with DAPI (*n* = 3). **l** Global m^6^A levels in JS-1 cells detected by high-performance liquid chromatography-tandem mass spectrometry (*n* = 3). **m **and** n** mRNA and protein expression levels of METTL3, METTL14, and FTO in JS-1 cells (*n* = 3). Data are presented as mean ± SEM; ^*^P < 0.05, ^**^P < 0.01

To investigate the role of m^6^A in AFB_1_-induced activation of HSC, we first isolated primary HSC from AFB_1_-treated mice that had undergone 21 days of gavage (Fig. 3a). Flow cytometry, utilizing the spontaneous blue fluorescence property of HSC, confirmed that the purity of the isolated primary cells was 95.88% (Fig. S3a). Compared to primary hepatocytes, these cells showed significantly higher expression of the HSC marker gene *Lrat* (Fig. S3b). Our results demonstrated that AFB_1_ significantly increased the expression of COL1A1 and α-SMA at both the gene and protein levels in primary HSC (*P* < 0.01; Fig. 3b, c and Fig. S3c). RNA sequencing of primary HSC from both control and AFB_1_-treated mice revealed distinct clustering of the two groups, as indicated by PCA analysis and differential expression volcano plots, and AFB_1_ treatment resulted in the upregulation of 753 genes and the downregulation of 1,399 genes (Fig. S3d and e). GO and KEGG pathway analyses showed that AFB_1_ significantly enriched pathways associated with HSC activation, including retinol metabolism, *PPAR* signaling, and ECM deposition (Fig. 3d and e). Additionally, AFB_1_ induced increased m^6^A modifications in primary HSC (*P* < 0.05; Fig. 3f), accompanied by a significant upregulation of METTL3 at both the gene and protein levels, and a notable downregulation of FTO (*P* < 0.05; Fig. 3g, h and Fig. S3f). Additionally, we observed in GSEA analysis that RNA methylation pathways were enriched in primary HSC (Fig. S3g). Among the enriched genes in this pathway, we identified significant upregulation of *METTL3* and *METTL14*, as well as a notable downregulation of *FTO* (*P* < 0.01; Fig. S3h).

In addition, we treated the mouse stellate cell line JS-1 with AFB_1_ in vitro and found that AFB_1_ also significantly upregulated COL1A1 and α-SMA at both the gene and protein levels (*P* < 0.01; Fig. 3i and j), which was further confirmed by immunofluorescence (Fig. 3k). AFB_1_ also increased m^6^A modifications in JS-1 cells (*P* < 0.01) (Fig. 3l). However, in contrast to primary HSC, AFB_1_ only elevated the gene and protein levels of METTL3 in JS-1 cells. Although AFB_1_ reduced *FTO* mRNA expression, it had no effect on FTO protein levels (*P* < 0.05; Fig. 3m, n and Fig. S3i).

### Inhibition of METTL3 alleviates AFB_1_-induced HSC activation

**Fig. 4:**
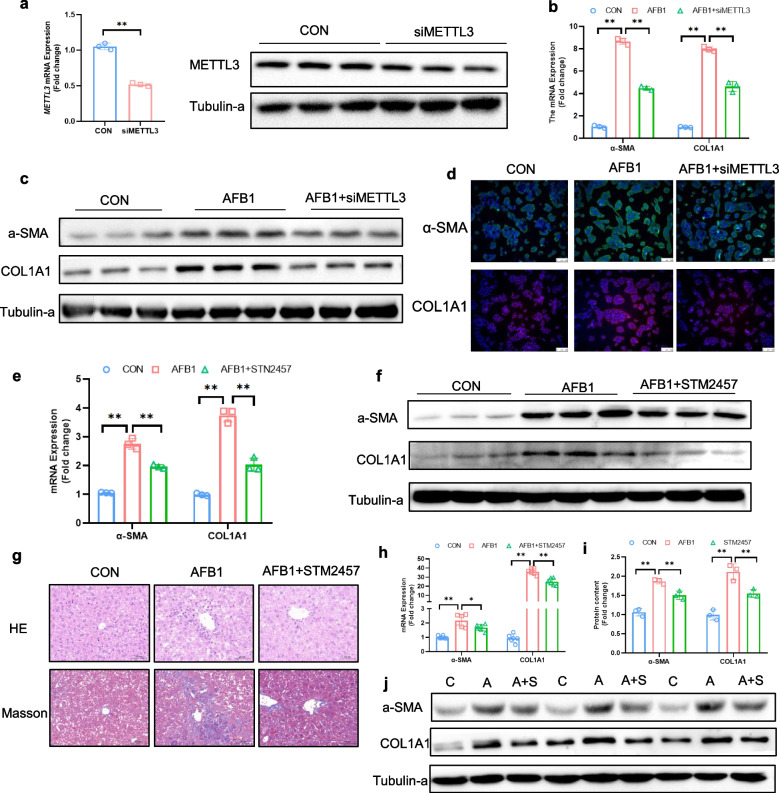
METTL3 Inhibition alleviates AFB_1_-induced HSC activation. **a** mRNA and protein expression levels of METTL3 in JS-1 cells (*n* = 3). **b **and** c** mRNA and protein expression levels of α-SMA and COL1A1 in JS-1 cells (*n* = 3). **d** Immunofluorescent staining of α-SMA and COL1A1 proteins. Nucleus was stained with DAPI (*n* = 3). **e **and** f** mRNA and protein expression levels of α-SMA and COL1A1 in primary HSC (*n* = 3). **g** HE and Masson staining of liver sections (*n* = 3). **h–j** mRNA and protein expression levels of α-SMA and COL1A1 in the liver (*n* = 3). Data are presented as mean ± SEM; ^*^P < 0.05, ^**^P < 0.01

siRNA targeting *METTL3* was synthesized to ensure significant knockdown of *METTL3* expression (*P* < 0.01; Fig. 4a and Fig. S4a). Our results demonstrated that silencing *METTL3* in JS-1 cells using siRNA markedly reduced the AFB_1_-induced upregulation of α-SMA and COL1A1 at both the mRNA and protein levels (*P* < 0.01; Fig. 4b, c and Fig. S4b). Immunofluorescence analysis further confirmed that *METTL3* knockdown significantly attenuated the AFB_1_-induced increase in α-SMA and COL1A1 fluorescence intensity (*P* < 0.01; Fig. 4d and Fig. S4c, d). These findings indicate that METTL3 silencing mitigates AFB_1_-induced JS-1 activation.

To further validate whether this effect extends in vivo, a METTL3 inhibitor (STM2457) was administered intraperitoneally to mice, and its impact on AFB_1_-induced primary HSC activation was assessed. The results showed that STM2457 effectively reduced AFB_1_-induced upregulation of α-SMA and COL1A1 at both the mRNA and protein levels in primary HSC (*P* < 0.05; Fig. 4e, f and Fig. S4e). Moreover, STM2457 alleviated AFB_1_-induced hepatic inflammatory infiltration and Masson’s blue staining enhancement (Fig. 4g), reduced AFB_1_-induced elevation of serum AST and ALT levels (Fig S4f), and diminished AFB_1_-induced upregulation of α-SMA and COL1A1 in the liver at both the mRNA and protein levels (*P* < 0.05; Fig. 4h–j). Collectively, these findings suggest that METTL3 inhibition attenuates AFB_1_-induced HSC activation and alleviates liver fibrosis.

### METTL3 knockdown reduces AFB_1_-induced m^6^A modification of Collagen mRNA and decreases its stability

**Fig. 5:**
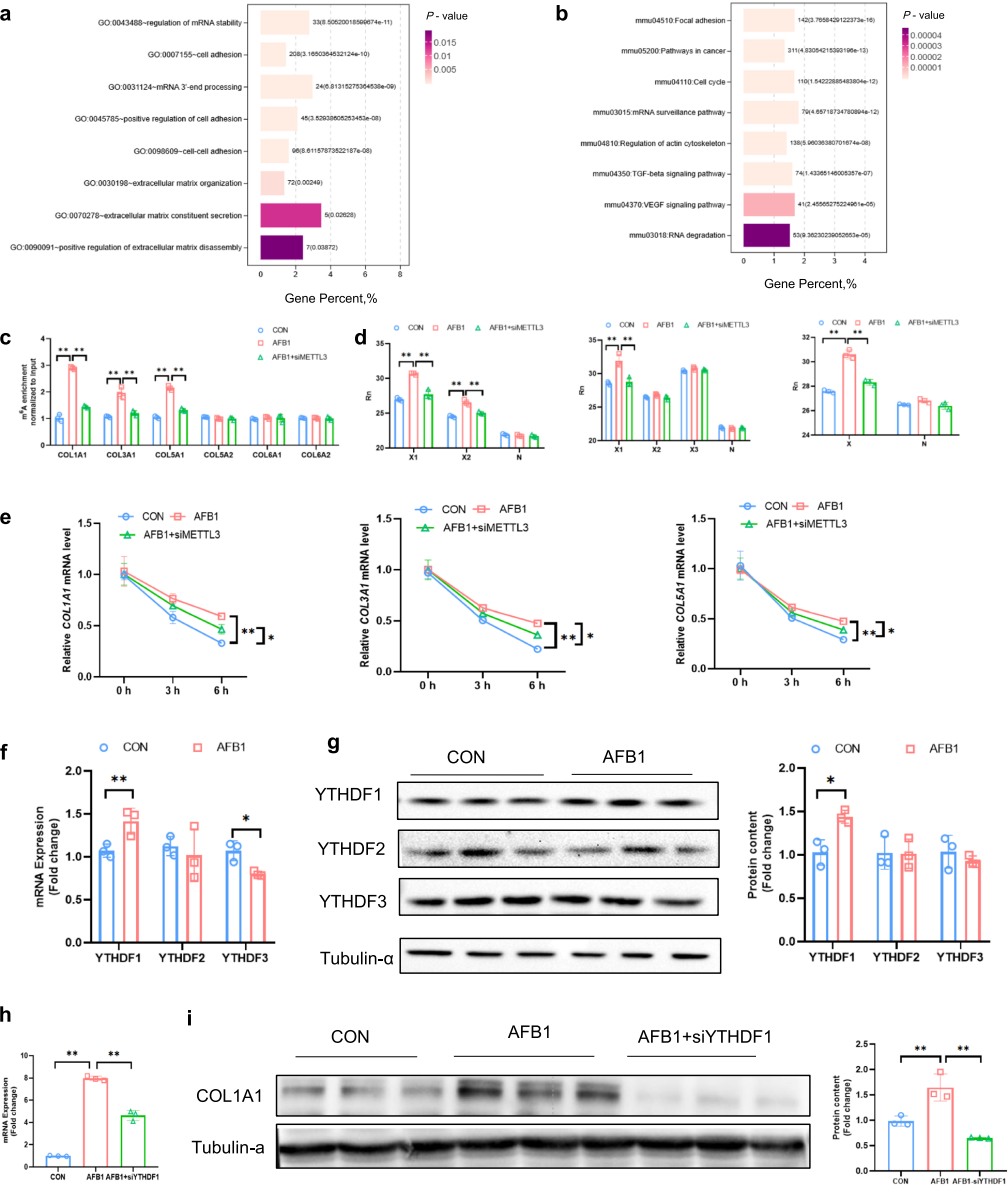
METTL3 knockdown decreases mRNA stability of collagens via m^6^A-mediated regulation. **a **and** b** GO and KEGG pathways of DEGs in HSC isolated from METTL3-cKO mice compared with wild-type mice. **c**. m^6^A abundance of collagens mRNA detected by MeRIP-PCR. **d** m^6^A modification in *COL1A1*, *COL3A1*, and *COL5A1* mRNAs in JS-1 cells detected with SELECT (*n* = 3). **e** Lifetime of *COL1A1*, *COL3A1* and *COL5A1* mRNAs in JS-1 cells (*n* = 3). **f** and **g** The mRNA and protein expression levels of YTHDF1/2/3 in JS-1 cells (*n* = 3). **h **and** i** mRNA and protein expression levels of COL1A1 in JS-1 cells (*n* = 3). Data are presented as mean ± SEM; ^*^P < 0.05, ^**^P < 0.01

In-depth analysis of publicly available raw data from the GSE207910 dataset revealed that METTL3 knockout significantly affected pathways related to RNA processing, stability, and 3’-end processing, as well as ECM secretion-associated pathways in HSC (Fig. 5a). Further KEGG pathway enrichment analysis demonstrated that METTL3 deletion profoundly impacted key pathways involved in RNA fate determination and stability in HSC (Fig. 5b). These findings suggest that METTL3 plays a crucial role in regulating RNA stability and ECM homeostasis in HSC. Further analysis of MeRIP-seq data revealed that METTL3 knockout significantly altered m^6^A peaks on transcripts encoding major ECM components, including *COL1A1*, *COL3A1*, *COL5A1*, *COL5A2*, *COL6A1*, and *COL6A2* (Fig. S5a–f). To validate whether METTL3 knockdown similarly affects AFB_1_-induced m^6^A modifications of Collagen mRNAs during HSC activation, MeRIP-PCR was performed. The results showed that METTL3 knockdown attenuated the AFB_1_-induced increase in m^6^A modifications on *COL1A1*, *COL3A1*, and *COL5A1* mRNAs (*P* < 0.01; Fig. 5c). Further validation using SELECT confirmed that METTL3 depletion significantly reduced AFB_1_-induced m^6^A methylation at specific sites within *COL1A1*, *COL3A1*, and *COL5A1* mRNAs (*P* < 0.01; Fig. 5d). Moreover, METTL3 knockdown markedly decreased AFB_1_-induced stabilization of *COL1A1*, *COL3A1*, and *COL5A1* mRNAs (*P* < 0.05; Fig. 5e). We next examined the expression of m^6^A reader proteins from the YTHDF family and found that AFB_1_ treatment upregulated YTHDF1 expression at both the mRNA and protein levels (*P* < 0.01, Fig. 5f and g). Notably, *YTHDF1* knockdown also mitigated AFB_1_-induced JS-1 activation (*P* < 0.05; Fig. 5h and i). These findings suggest that m^6^A modifications on Collagen mRNAs are critical for AFB_1_-induced HSC activation. METTL3 depletion reduces m^6^A modifications on Collagen mRNAs, thereby decreasing their stability and limiting ECM component accumulation.

### Sitagliptin alleviates AFB_1_-induced liver fibrosis in mice by targeting METTL3

**Fig. 6:**
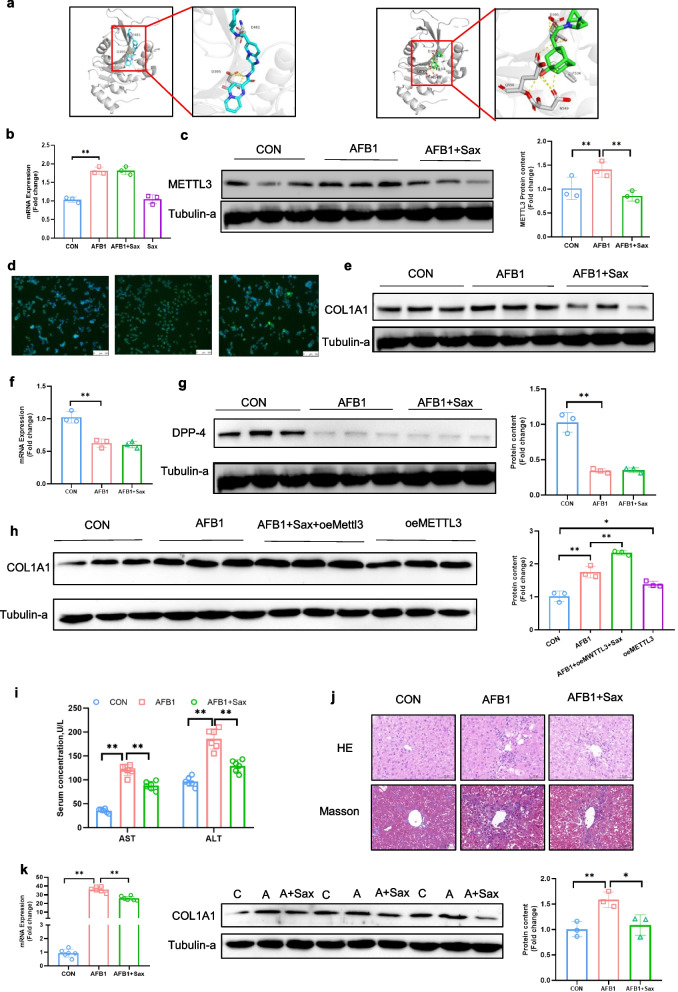
Sitagliptin alleviates AFB_1_-induced liver fibrosis in mice by targeting METTL3. **a** Molecular docking of METTL3-inhibitor STM2457 and saxagliptin with METTL3. **b **and** c** mRNA and protein expression levels of METTL3 in JS-1 cells (*n* = 3). **d** Immunofluorescent staining of METTL3 protein. Nucleus was stained with DAPI (*n* = 3). **e** Protein expression levels of COL1A1 in JS-1 cells (*n* = 3). **f **and **g** mRNA and protein expression levels of DPP-4 in JS-1 cells (*n* = 3). **h** Protein expression levels of COL1A1 in JS-1 cells (*n* = 3). **i** Plasma AST and ALT activities (*n* = 6). **j** HE and Masson staining of liver sections (*n* = 3). **k **and** l** Hepatic mRNA and protein expression levels of α-SMA and COL1A1 (*n* = 3). Data are presented as mean ± SEM; ^*^P < 0.05, ^**^P < 0.01

Molecular docking was performed to evaluate the interaction between saxagliptin and METTL3, using the METTL3 inhibitor STM2457 as a positive control (Fig. 6a). The docking results showed that STM2457 bound to METTL3 with a binding energy of −8.557 kcal/mol, whereas saxagliptin exhibited a binding energy of −7.067 kcal/mol. Further analysis revealed that STM2457 interacted with METTL3 at residues E481 and D395, while saxagliptin formed interactions with D395, F534, Q550, and N549 (Table 1). Notably, D395 plays a critical role in the methyltransferase activity of METTL3. Treatment of JS-1 cells with saxagliptin did not affect AFB_1_-induced *METTL3* upregulation at the mRNA level (*P* < 0.01; Fig. 6b), but significantly reduced AFB_1_-induced METTL3 protein expression (*P* < 0.01; Fig. 6c). Immunofluorescence analysis further confirmed that saxagliptin attenuated the AFB_1_-induced increase in METTL3 fluorescence intensity (Fig. 6d). Moreover, saxagliptin treatment reduced AFB_1_-induced COL1A1 expression at both the mRNA and protein levels (*P* < 0.01; Fig. 6e and Fig S6a, b). Since saxagliptin is clinically used as a *DPP-4* inhibitor, we examined whether its effects were mediated through *DPP-4*. However, our results showed that saxagliptin did not reverse AFB_1_-induced downregulation of DPP-4 at either the mRNA or protein level (Fig. 6f and g). Furthermore, *METTL3* overexpression abolished the protective effects of saxagliptin against AFB_1_-induced *COL1A1* upregulation (*P* < 0.01; Fig. 6h), suggesting that saxagliptin directly targets *METTL3* to mitigate *COL1A1* secretion. Finally, oral administration of saxagliptin significantly reduced AFB_1_-induced increases in serum AST and ALT levels (Fig. 6i), attenuated hepatic inflammatory infiltration and Masson’s blue staining (*P* < 0.01; Fig. 6j), and decreased AFB_1_-induced COL1A1 expression at both the mRNA and protein levels in liver tissue (*P* < 0.01; Fig. 6k and l). In addition, saxagliptin effectively reduced AFB_1_-induced upregulation of α-SMA and COL1A1 at both the mRNA and protein levels in primary HSC (*P* < 0.05; Fig. S6c–e).

**Table 1:** Binding energies and binding sites of STM2457 and saxagliptin with METTL3

Name	Binding energy with METTL3, kcal/mol	Binding site to METTL3
STM2457	−8.557	E481, D395
Saxagliptin	−7.067	D395, F534, Q550, N549

## Discussion

This study systematically investigates the hepatotoxic effects of AFB_1_ in a mouse model, with a particular focus on its role in liver fibrosis progression. Our findings reveal that AFB_1_ exposure induces significant alterations in m^6^A RNA modification, which in turn modulates HSC activation and contributes to fibrosis. While extensive research has established that AFB_1_-induced liver damage is mediated through inflammation, oxidative stress, and various signaling pathways [21], its impact on epigenetic regulation, particularly m^6^A modification, in HSC activation and fibrosis remains largely unexplored. Here, we address this gap by demonstrating that AFB_1_ exposure alters m^6^A methylation patterns, thereby influencing liver fibrosis progression.

AFB_1_-induced liver toxicity is multifaceted, involving direct hepatocellular damage as well as secondary disruptions in metabolic homeostasis, oxidative stress, and mitochondrial apoptosis [22–24]. Moreover, AFB_1_ exposure significantly enhances inflammation and promotes the expression of pro-fibrotic factors, driving the fibrotic cascade [25]. Our in vivo results show that AFB_1_ exposure leads to reduced body weight and food intake in mice, an increased liver-to-body weight ratio, and elevated serum AST and ALT levels, collectively indicating AFB_1_-induced liver injury. Additionally, AFB_1_ significantly upregulated inflammatory cytokines IL-1β, IL-6 and the pro-fibrotic factor TGF-β, along with key fibrosis markers α-SMA and COL1A1, further confirming its role in driving liver fibrosis.

HSC activation is a pivotal event in fibrosis progression. Upon activation, HSC transdifferentiate into MFCs, secreting large quantities of collagen and ECM components, thereby disrupting normal liver architecture and exacerbating hepatic dysfunction [26, 27]. While previous studies have largely focused on indirect mechanisms of AFB_1_-induced HSC activation—such as the activation of the p53-Parkin-dependent mitophagy pathway in hepatocytes and subsequent release of CD63-containing EVs [13]. However, studies investigating whether AFB_1_ can directly activate HSC remain limited. Our study provides novel evidence that AFB_1_ directly induces HSC activation, as indicated by the significant upregulation of α-SMA and COL1A1 expression in both primary HSC and the JS-1 cell line, confirming its direct pro-fibrotic role.

Epigenetic modifications, including DNA methylation, noncoding RNA regulation, and histone modifications, have been implicated in AFB_1_-induced toxicity and various human diseases [28]. Increasing evidence highlights the crucial role of m^6^A modification in liver disease progression [29], particularly in HSC activation. For instance, the m^6^A demethylase ALKBH5 modulates Drp1 methylation to regulate mitochondrial fission and alleviate fibrosis [30]. In our study, we observed that m^6^A levels were significantly elevated in AFB_1_-induced HSC activation. Further analysis revealed that AFB_1_ upregulated METTL3 while downregulating FTO, leading to an overall increase in m^6^A modification. snRNA-seq further revealed that AFB_1_ exposure significantly altered the transcriptional profile of HSC, with aHSC exhibiting increased METTL3 and METTL14 expression, while qHSC showed higher FTO expression. This suggests that m^6^A methyltransferases and demethylases regulate distinct stages of HSC activation, underscoring the critical role of dynamic m^6^A modification in fibrosis progression.

As the primary m^6^A methyltransferase, METTL3 plays a crucial role in fibrosis regulation [31]. Given that METTL3 has been shown to enhance TGF-β-induced HSC activation and contribute to macrophage pyroptosis and M1 polarization in liver fibrosis [32, 33]. Our findings further support its pro-fibrotic role. Importantly, both in vivo and in vitro inhibition of METTL3 effectively suppressed AFB_1_-induced HSC activation and attenuated fibrosis, underscoring its potential as a therapeutic target. Previous studies have demonstrated that METTL3-mediated m^6^A methylation of miR-350 promotes liver fibrosis by downregulating SPRY2, thereby activating the PI3K/AKT and ERK pathways [34]. Additionally, METTL3-driven NR1D1 ablation disrupts HSC circadian rhythms, exacerbating fibrosis [35], while METTL3-mediated regulation of THY1 enhances HSC activation and fibrosis. [36]. Consistent with these findings, our study confirms that METTL3 inhibition mitigates AFB_1_-induced HSC activation and fibrosis. Moreover, we identified saxagliptin, a clinically available small-molecule drug, as a potent METTL3 inhibitor that significantly reduces AFB_1_-induced HSC activation and alleviates liver fibrosis in mice. These findings suggest that targeting METTL3 may provide a promising therapeutic strategy for AFB_1_-induced liver fibrosis.

## Conclusion

**Fig. 7:**
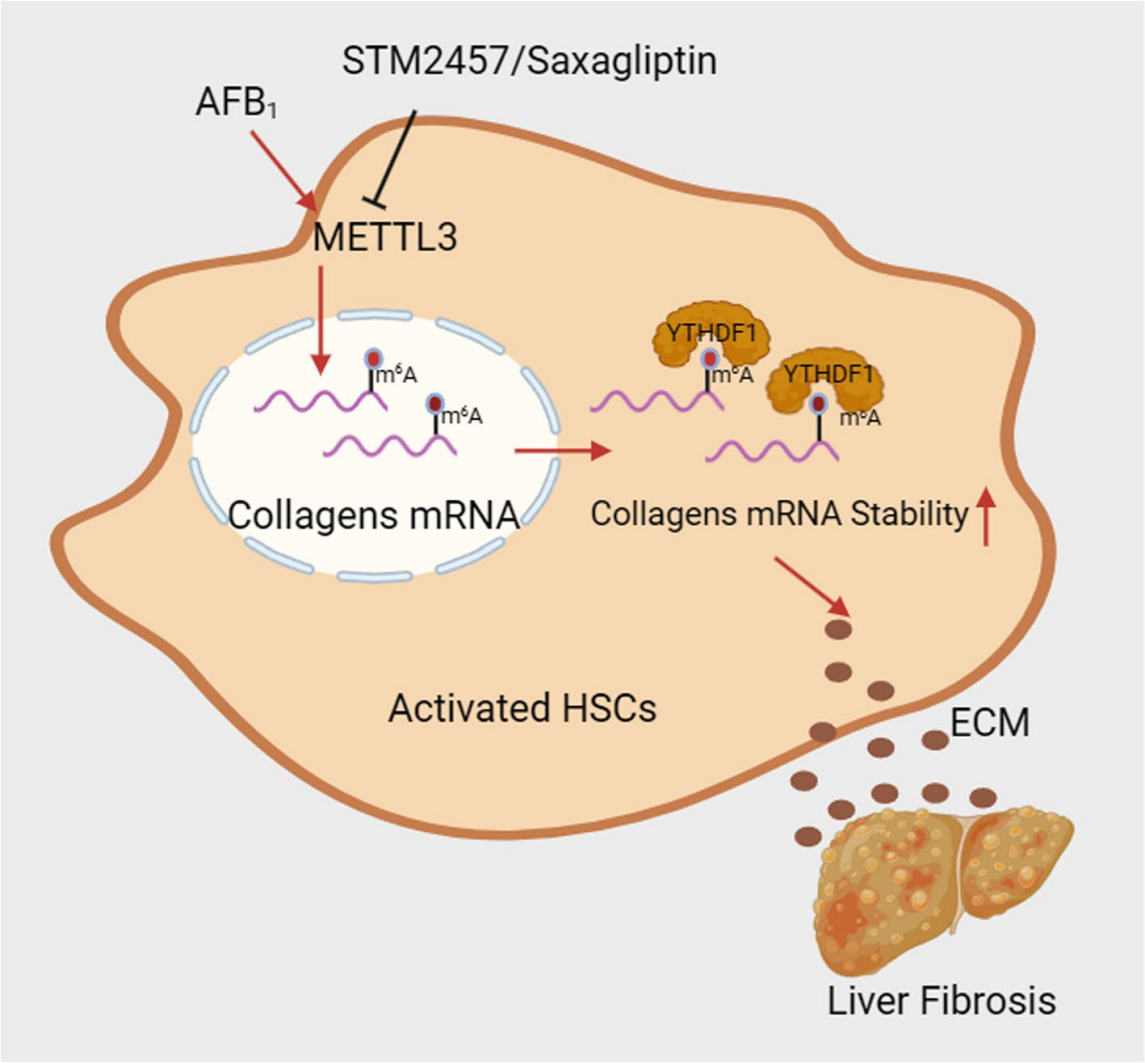
Graphic overview

This study provides direct evidence that AFB_1_ promotes HSC activation and liver fibrosis via m^6^A RNA modification, with the overall mechanistic framework summarized in Fig. 7. Our findings delineate a critical role for METTL3 in mediating AFB_1_-induced fibrosis and propose METTL3 as a potential therapeutic target. These results underscore the importance of epitranscriptomic regulation in environmental toxin-induced liver pathology and warrant further investigation into m^6^A-targeted strategies for the prevention and treatment of AFB_1_-associated hepatic disorders.

## Supplementary Information

Additional file 1: Fig. S1. Hepatic fibrosis caused by AFB_1_ is associated with global RNA m^6^A hypermethylation. Fig. S2. Hepatic fibrosis caused by AFB_1_ is associated with global RNA m^6^A hypermethylation. Fig. S3. AFB_1_ activates primary HSC and JS-1 cells, accompanied by an increase in m^6^A modifications. Fig. S4. Inhibition of METTL3 alleviates AFB_1_-induced HSC activation. Fig. S5. METTL3 knockdown reduces AFB_1_-induced m^6^A modification of Collagen mRNA and decreases its stability. Fig. S6. Sitagliptin targeting METTL3 alleviates AFB_1_-induced liver fibrosis in mice. Table S1. Nucleotide sequences of primers. Table S2. The list of antibodies. Table S3. Nucleotide sequences of primers for MeRIP-PCR. Table S4. Nucleotide sequences of SELECT method.

Additional file 2: Original Western blot images.

## Data Availability

RNA-seq data have not yet been uploaded to a public database but are available upon request from the corresponding author (zhaoruqian@njau.edu.cn). The MeRIP-seq dataset is accessible under the accession number GSE207910.

## Abbreviations

α-SMA: α Smooth muscle actin
Act-D: Actinomycin D
ALKBH5: AlkB homolog 5
ALT: Alanine aminotransferase
AST: Aspartate aminotransferase
CCl4: Carbon tetrachloride
COL1A1: Collagen type I alpha 1
Cyc: Cycloleucine
DAMP: Damage-associated molecular patterns
ECM: Extracellular matrix
FTO: Fat mass and obesity-associated protein
HSC: Hepatic stellate cells
METTL3: Methyltransferase 3
METTL14: Methyltransferase 14
YTHDFs: YTH domain family proteins
