# Supplementary material for: METTL3 inhibition attenuates AFB1-induced hepatic fibrosis by suppressing m6A-mediated hepatic stellate cell activation
Source: J Anim Sci Biotechnol. 2026 May 28;17:102. doi: 10.1186/s40104-026-01380-4 (PMC13217767; doi:10.1186/s40104-026-01380-4)
Supplement: Supplementary file 1 — Additional file 1: Fig. S1. Hepatic fibrosis caused by AFB1 is associated with global RNA m6A hypermethylation. Fig. S2. Hepatic fibrosis caused by AFB1 is associated with global RNA m6A hypermethylation. Fig. S3. AFB1 activates primary HSC and JS-1 cells, accompanied by an increase in m6A modifications. Fig. S4. Inhibition of METTL3 alleviates AFB1-induced HSC activation. Fig. S5. METTL3 knockdown reduces AFB1-induced m6A modification of Collagen mRNA and decreases its stability. Fig. S6. Sitagliptin targeting METTL3 alleviates AFB1-induced liver fibrosis in mice. Table S1. Nucleotide sequences of primers. Table S2. The list of antibodies. Table S3. Nucleotide sequences of primers for MeRIP-PCR. Table S4. Nucleotide sequences of SELECT method. [file 40104_2026_1380_MOESM1_ESM.docx]

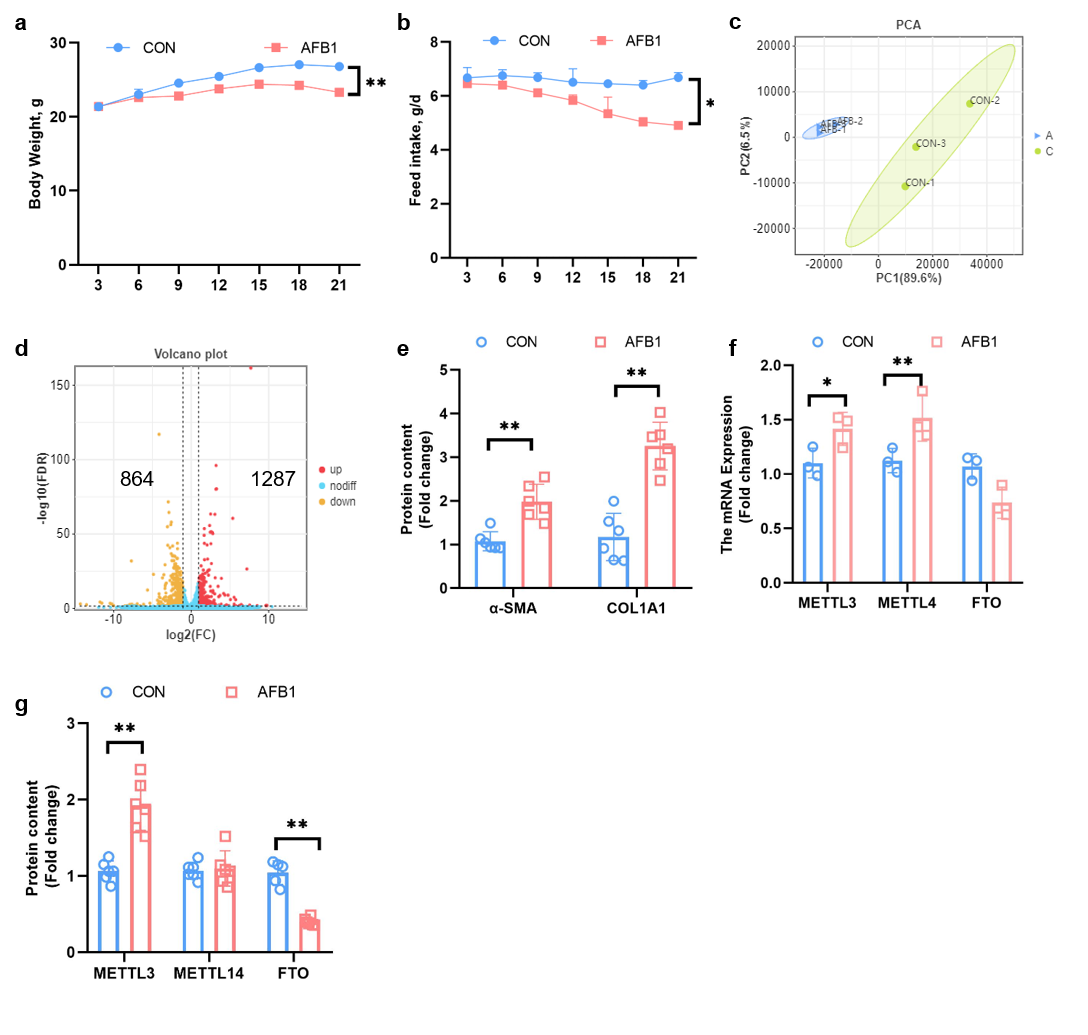


**Fig. S1** Hepatic fibrosis caused by AFB_1_ is associated with global RNA m^6^A hypermethylation. **A** and **b** Body weight and feed intake of CON and AFB1 mice at indicated time points (*n* = 6). **c** PCA analysis plot in liver (*n* = 3). **d** Differential gene scatter plot in liver (*n* = 3). **e** Protein expression of α-SMA and COL1A1 in the liver (*n* = 6). **F** and **g** mRNA and protein expression of METTL3, METTL14 and FTO in the liver (*n* = 6). Data are presented as mean ± SE; ^*^*P* < 0.05, ^**^*P* < 0.01


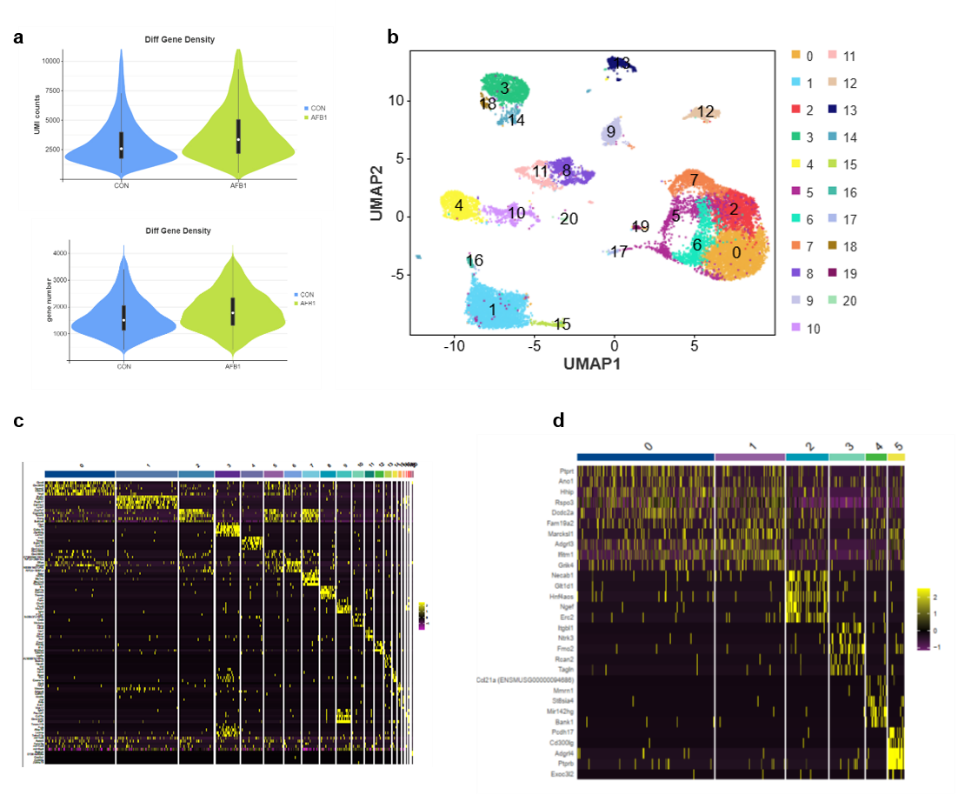


**Fig. S2** SnRNA-seq reveals AFB_1_ alters m^6^A methyltransferase and demethylase distribution in HSC. **a** Violin plot showing the number of UMAPs and captured genes for different snRNA-seq libraries. **b** UMAP showing the clustering results of snRNA-seq for liver samples from different treatments. **c** Heatmap depicting the top 50 differentially expressed genes for cell types. **d** Heatmap depicting the top 50 differentially expressed genes for qHSC and aHSC


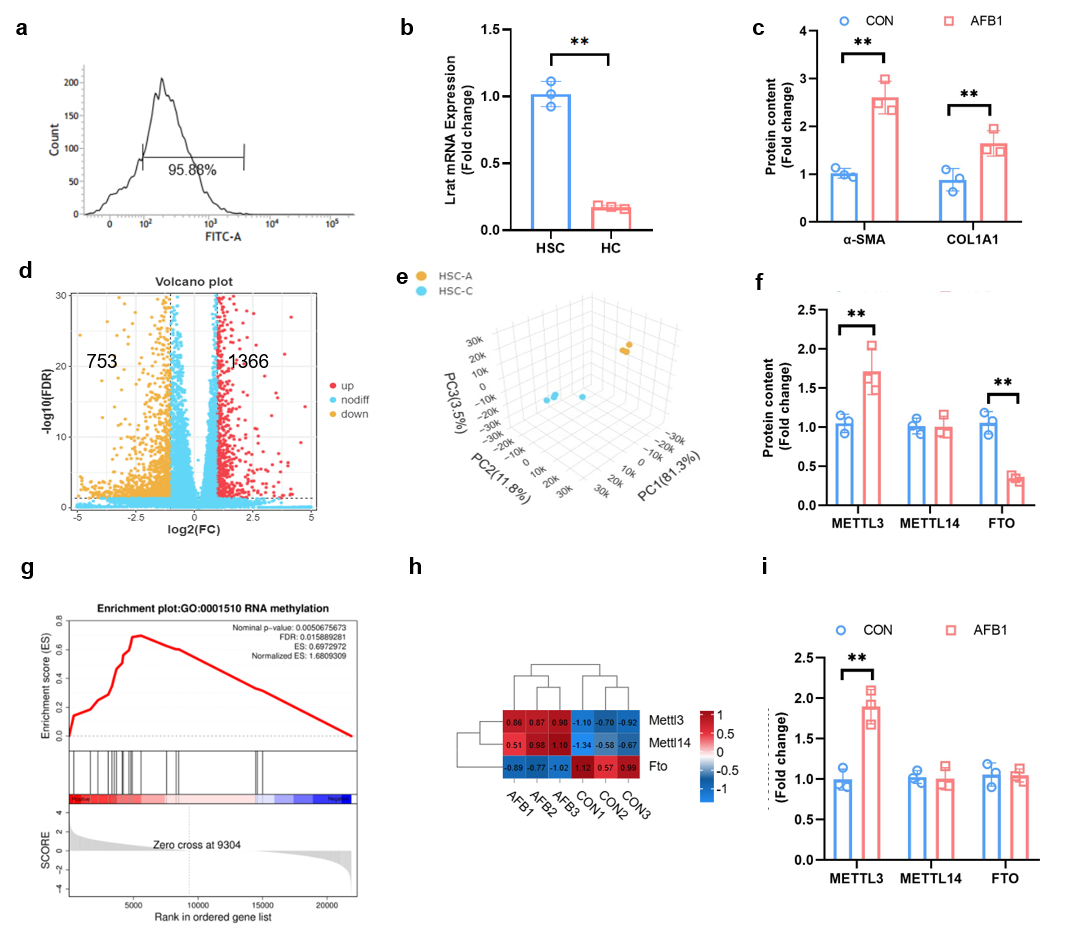


**Fig. S3** AFB_1_ activates primary HSC and JS-1 cells, accompanied by an increase in m^6^A modifications. **a** Flow cytometry for detecting the purity of primary HSC. **b** mRNA expression levels of Lrat in primary HSC (*n* = 3). **c** Protein expression levels of α-SMA and COL1A1 in JS-1 (*n* = 3). **d** PCA analysis plot in primary HSC (*n* = 3). **e** Differential gene scatter plot in primary HSCs (*n* = 3). **f** GSEA analysis of RNA-seq data in primary HSC (*n* = 3). **g** Heatmap of m^6^A-related transcripts from RNA-seq data in primary HSC (*n* = 3). **h** Protein expression of METTL3, METTL14 and FTO in primary HSC (*n* = 3). Data are presented as mean ± SE; ^*^*P* < 0.05, ^**^*P* < 0.01


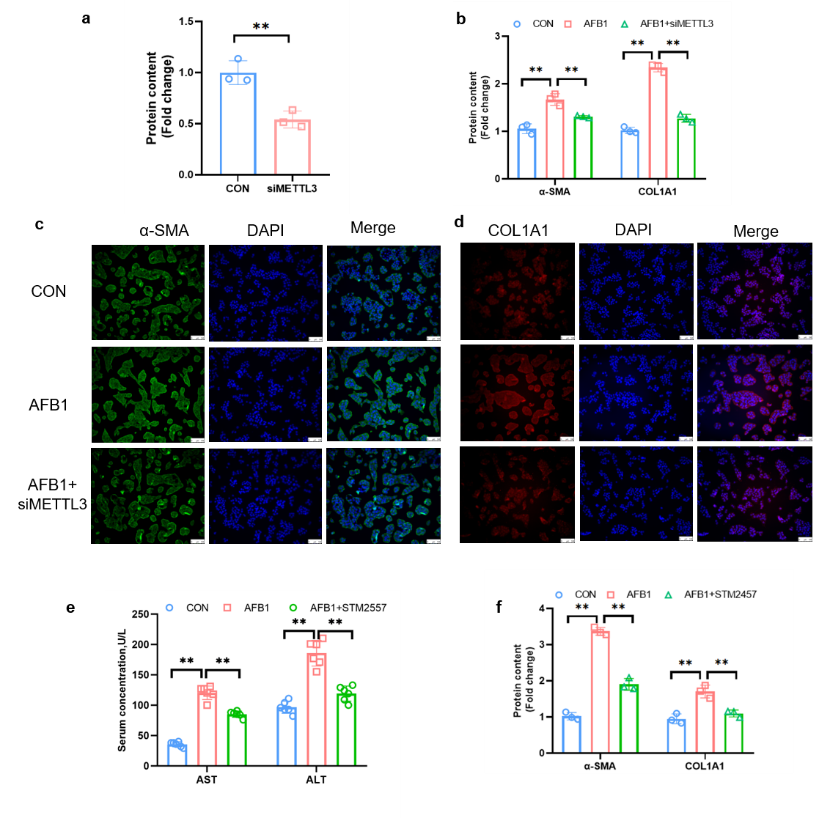


**Fig. S4** Inhibition of METTL3 alleviates AFB_1_-induced HSC activation. **a** Protein expression of METTL3 in JS-1 (*n* = 3). **b** Protein expression of α-SMA and COL1A1 in JS-1 (*n* = 3). **C** and **d** α-SMA and COL1A1 expression were assessed by immunofluorescence. Nucleus was stained with DAPI; magnification = 20× (*n* = 3). **e** AST and alanine ALT activities in plasma (*n* = 6). **f** Protein expression of α-SMA and COL1A1 in primary HSC (*n* = 3). Data are presented as mean ± SE; ^*^*P* < 0.05, ^**^*P* < 0.01


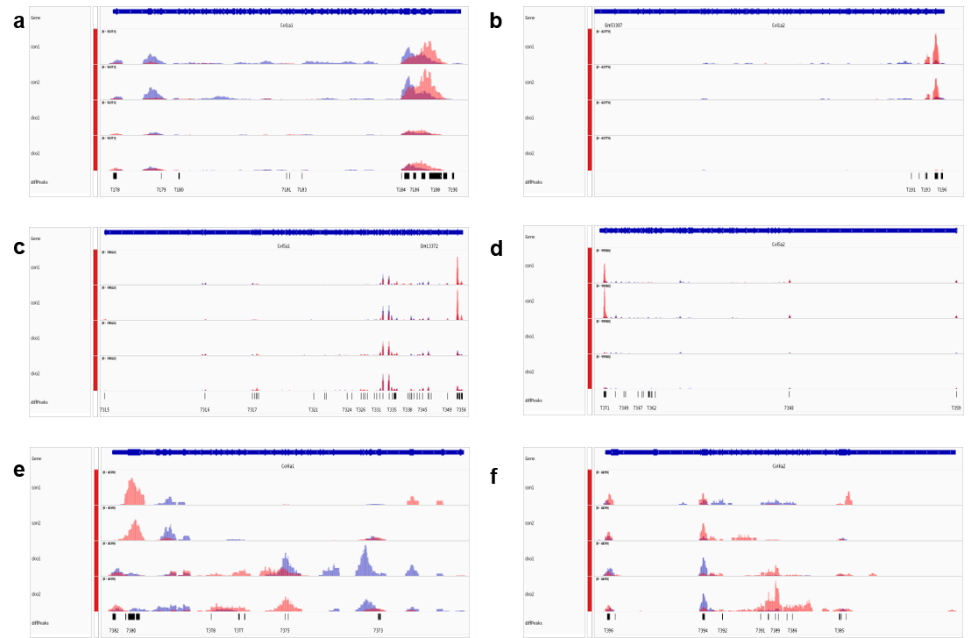


**Fig. S5** METTL3 knockdown reduces AFB_1_-induced m^6^A modification of Collagen mRNA and decreases its stability. **a-f** m^6^A peak marked in red is increased in *COL1A1*, *COL3A1*, *COL5A1*, *COL5A2*, *COL6A1*and *COL6A2* genes from m^6^A-seq data in the AFB_1_ group. Coverage of m^6^A immunoprecipitation (IP) and control reads (Input) are indicated in blue and gray, respectively


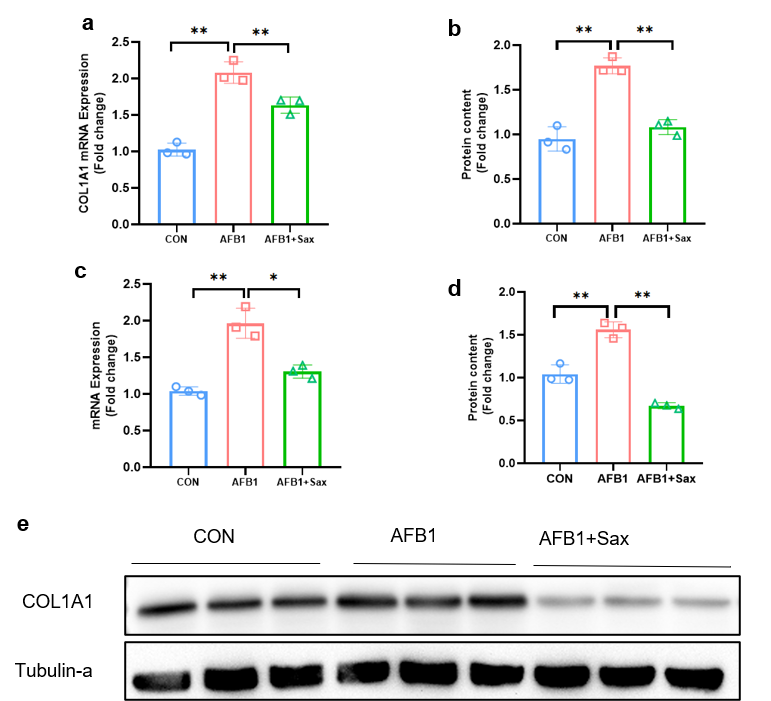


**Fig. S6** Sitagliptin targeting METTL3 alleviates AFB_1_-induced liver fibrosis in mice. **a** and **b** mRNA and protein expression of METTL3 in JS-1 (*n* = 3). **c** and **d** mRNA and protein expression of METTL3 in JS-1 (*n* = 3) . **e** Protein expression of COL1A1 in primary HSCs (*n* = 3). Data are presented as mean ± SE; ^*^*P* < 0.05, ^**^*P* < 0.01

**Table S1** Nucleotide sequences of primers

| **Target genes** | **Primer sequences (5’ to 3’)** | **Used for** |
| --- | --- | --- |
| *IL-1β* | F: AATGCCACCTTTTGACAGTGATG  R: GGAAGGTCCACGGGAAAGA | Real-time PCR |
| *IL-6* | F: GGAAGGTCCACGGGAAAGAC  R: GGAAGGTCCACGGGAAAGAC | Real-time PCR |
| *TGF-β1* | F: ACTGGAGTTGTACGGCAGTG  R: GGATCCACTTCCAACCCAGG | Real-time PCR |
| *Collagen I* | F: AGCACGTCTGGTTTGGAGAG  R: GCTGTAGGTGAAGCGACTGT | Real-time PCR |
| *α-SMA* | F: AAGCGAGGTATCCTGACCCT  R: GATGTCGCGCACAATCTCAC | Real-time PCR |
| *METTL3* | F: GCTCCATCCAGGCCCATAAG  R: CCCACTCACCGTATCGATGG | Real-time PCR |
| *METTL14* | F: GTGATTCTCCTGGAGCCACC  R: TGGGGTCCAGAGTCTTCGTT | Real-time PCR |
| *FTO* | F: TGAAGGTAGCGTGGGACATAGA  R: TGAAGGTAGCGTGGGACATAGA | Real-time PCR |
| *GAPDH* | F: TCTCCTGCGACTTCAACA  R: TGTAGCCGTATTCATTGTCA | Real-time PCR |

**Table S2** The list of antibodies

| **Antibodies** | **Source** | **Catalogue No.** | **Dilution** |
| --- | --- | --- | --- |
| COL1A1 | Boster | BA0325 | 1:500 |
| α-SMA | Proteintech | 14395-1-AP | 1:3000 |
| METTL3 | Abcam | AB98009 | 1:3000 |
| METTL14 | Abcam | AB98116 | 1:1000 |
| FTO | Abcam | AB77547 | 1:1000 |
| Tubulin-α | Bioworld | BS1699 | 1:5000 |

**Table S3** Nucleotide sequences of primers for MeRIP-PCR

| **Target genes** | **Primer sequences (5’ to 3’)** | **Used for** |
| --- | --- | --- |
| *COL1A1* | F: TCCATGGCCTCTGCAACAAAC | MeRIP-PCR |
|  | R: TGCACTGAGGAATAGAACGGT |  |
| *COL3A1* | F: CCAATGCTTGAGGGCAAGGT | MeRIP-PCR |
|  | R: CTCTCCTTCGTCTTCCTCACG |  |
| *COL5A1* | F: GGCGGGACAGTATTTGAAGA | MeRIP-PCR |
|  | R: GTGGATGTGTTGAGGGTTGT |  |
| *COL5A2* | F: GATGGTCATTGTCATTGGTTGCT | MeRIP-PCR |
|  | R: TGGGCCAGTTTGTTTCATGT |  |
| *COL6A1* | F: AGACGGTACACTTGGGTCTTT | MeRIP-PCR |
|  | R: GAAACAGGCAGTGGGAAGACT |  |
| *COL6A2* | F: AGGAGAAGTACCGTTAGCCAG | MeRIP-PCR |
|  | R: TACCGGGCTTTCCAATTCCTG |  |

**Table S4** Nucleotide sequences of SELECT method

| **Target** | **Sequences (5’ to 3’)** |
| --- | --- |
| COL1A1  X1 site | Up Probe: tagccagtaccgtagtgcgtgCCGATGTTTCCAGTCTGCTG |
|  | Down Probe: ACCCAGCCTGCACATGCATGcagaggctgagtcgctgcat |
| COL1A1  X2 site | Up Probe: tagccagtaccgtagtgcgtgTCACTTATTTGAAACAGACG |
|  | Down Probe: GGTTTTTAATTTAGGTTGAGcagaggctgagtcgctgcat |
| COL1A1  N site | Up Probe: tagccagtaccgtagtgcgtgGGCTCCAAACCCCTGAGAGGGG |
|  | Down Probe: CAGTCTGCTGTGACCCTGCCcagaggctgagtcgctgcat |
| COL3A1  X1 site | Up Probe: tagccagtaccgtagtgcgtgTCACTTATTTGAAACAGACG |
|  | Down Probe: GGTTTTTAATTTAGGTTGAGcagaggctgagtcgctgcat |
| COL3A1  X2 site | Up Probe: tagccagtaccgtagtgcgtgCTGGCCCAGCCCAGCTTCT |
|  | Down Probe: CGGGAGAAAGACTTTGACAGcagaggctgagtcgctgcat |
| COL3A1  X3 site | Up Probe: tagccagtaccgtagtgcgtgGACTTATATCTAAGCATCGG |
|  | Down Probe: TCTTCTTTCTTAATGTTATTcagaggctgagtcgctgcat |
| COL3A1  N site | Up Probe: tagccagtaccgtagtgcgtgATGGCGATGAGAAGCCGTGC |
|  | Down Probe: CAGACCAGTATAGCTGCACCcagaggctgagtcgctgcat |
| COL5A1  X site | Up Probe: tagccagtaccgtagtgcgtgTCATCCTGAGACTCTTGAAGT |
|  | Down Probe: AGAACTGTGACTGTTTAAAGcagaggctgagtcgctgcat |
| COL5A1  N site | Up Probe: tagccagtaccgtagtgcgtgACGATCTGAAGTGCCTGACC |
|  | Down Probe: TCCTGCATCAGCTTTGTACAcagaggctgagtcgctgcat |
| qPCR | Forward Prime: ATGCAGCGACTCAGCCTCTG |
|  | Reverse Prime: TAGCCAGTACCGTAGTGCGTG |
