# Supplementary material for: METTL3 inhibition attenuates AFB1-induced hepatic fibrosis by suppressing m6A-mediated hepatic stellate cell activation
Source: J Anim Sci Biotechnol. 2026 May 28;17:102. doi: 10.1186/s40104-026-01380-4 (PMC13217767; doi:10.1186/s40104-026-01380-4)

**Original WB images**

**Fig. 1**

COL1A1


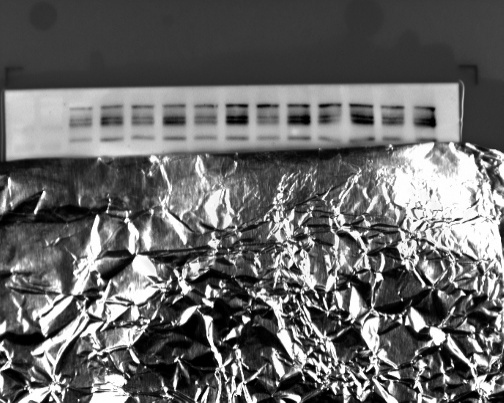


α-SMA


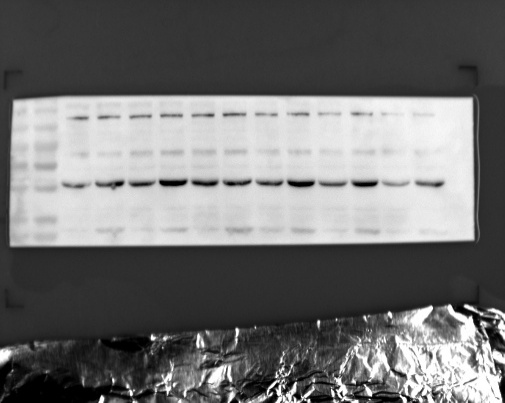


METTL3


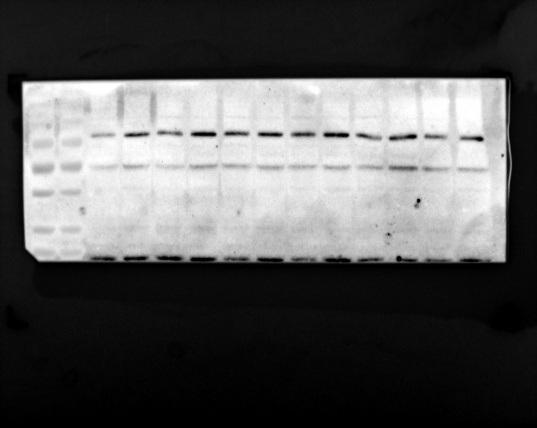


METTL14


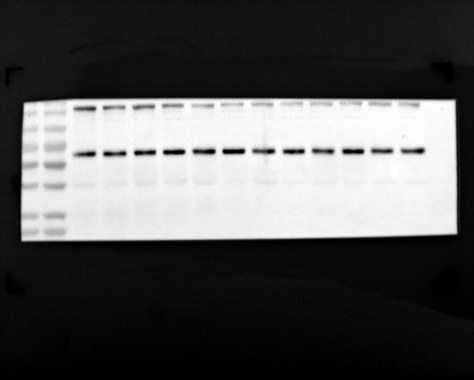


FTO


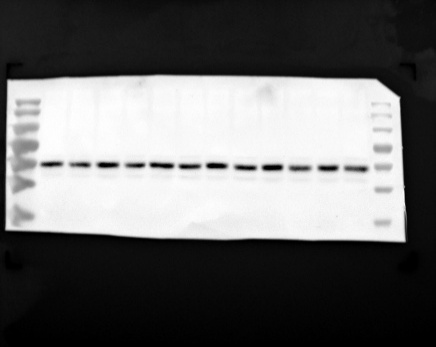


GAPDH


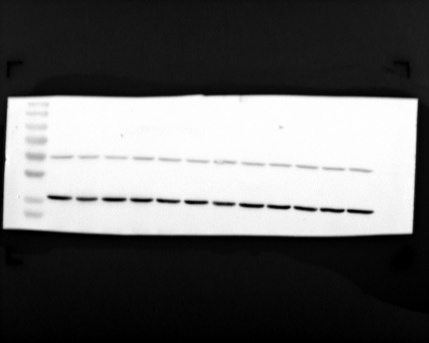


**Fig. 3**

α-SMA-1 HSC


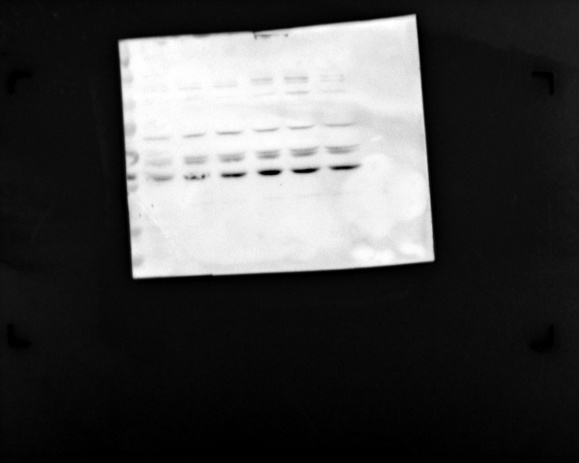


COL1A1-1 HSC


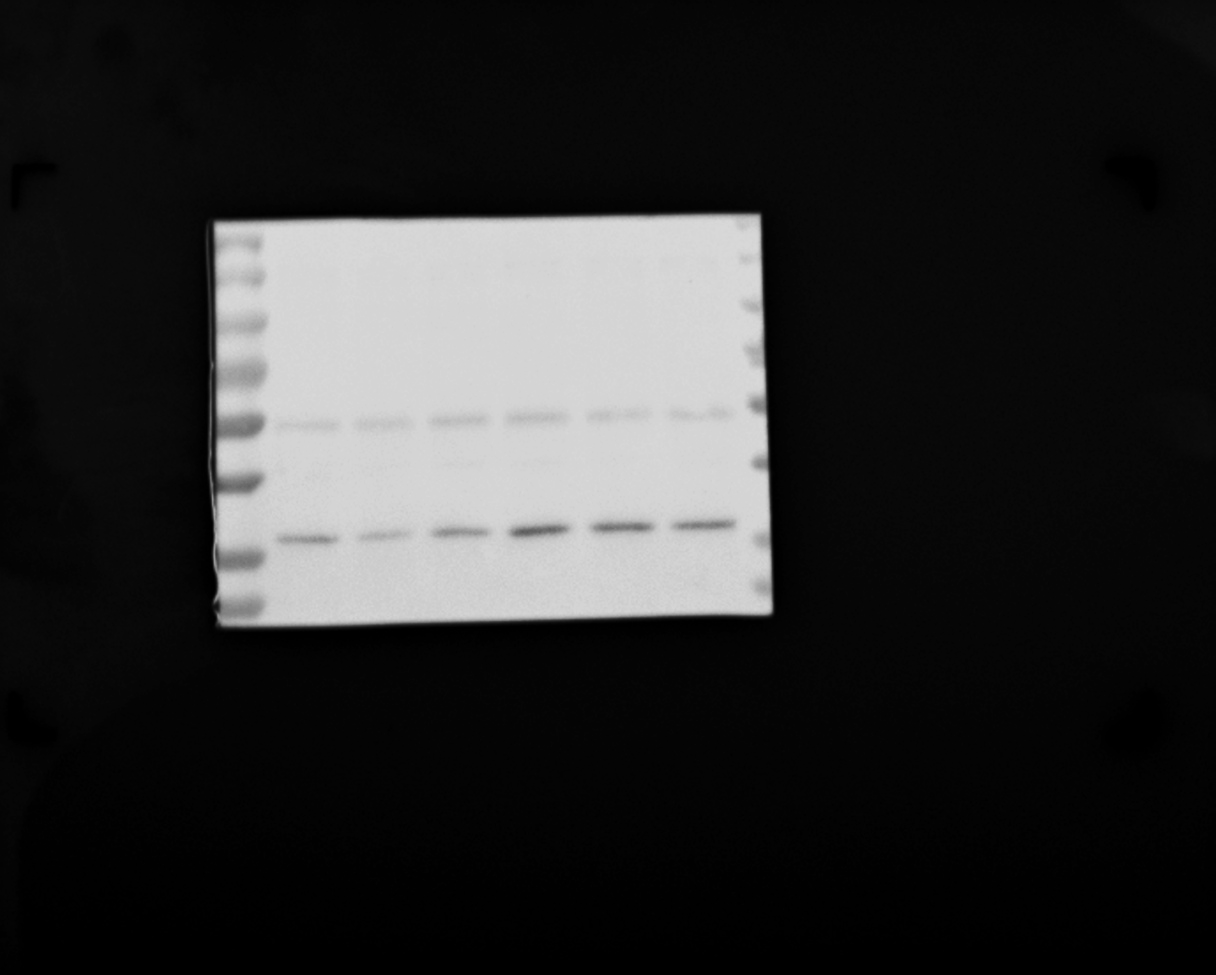


GAPDH HSC


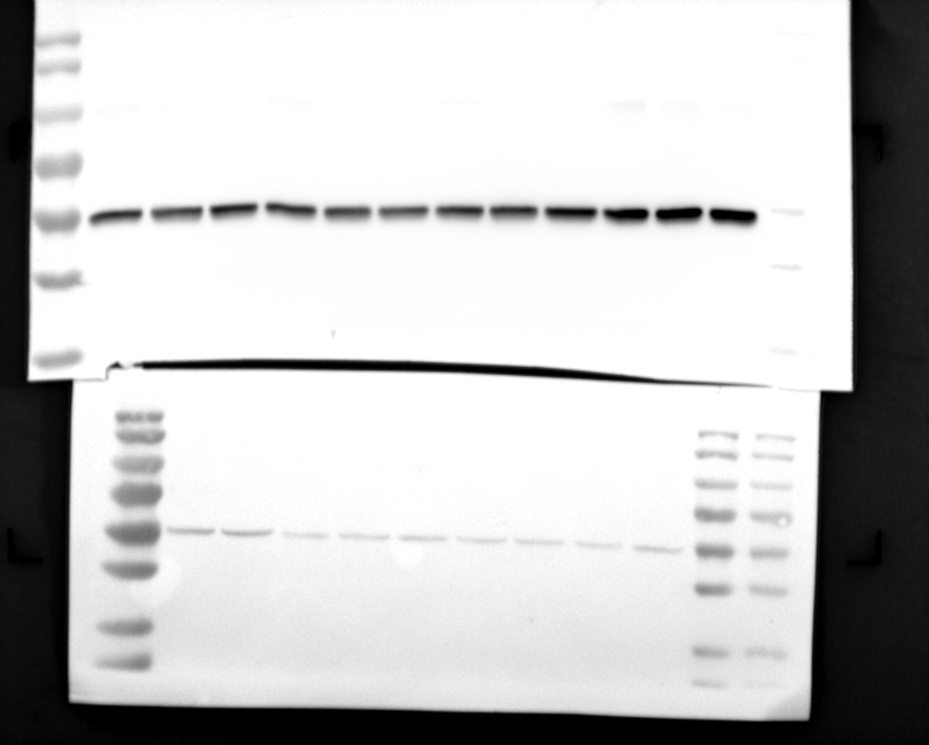


METTL3 HSC


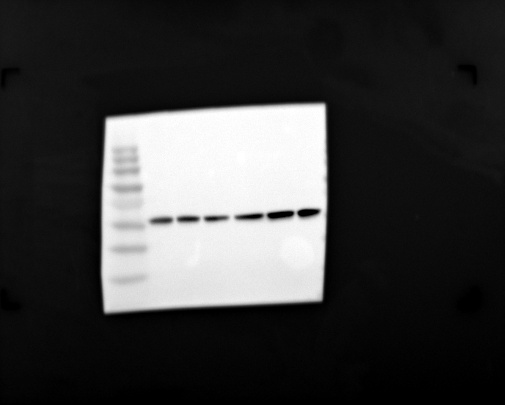


METTL14 HSC


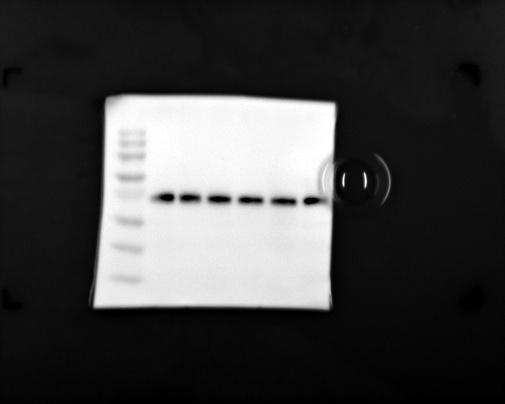


FTO HSC


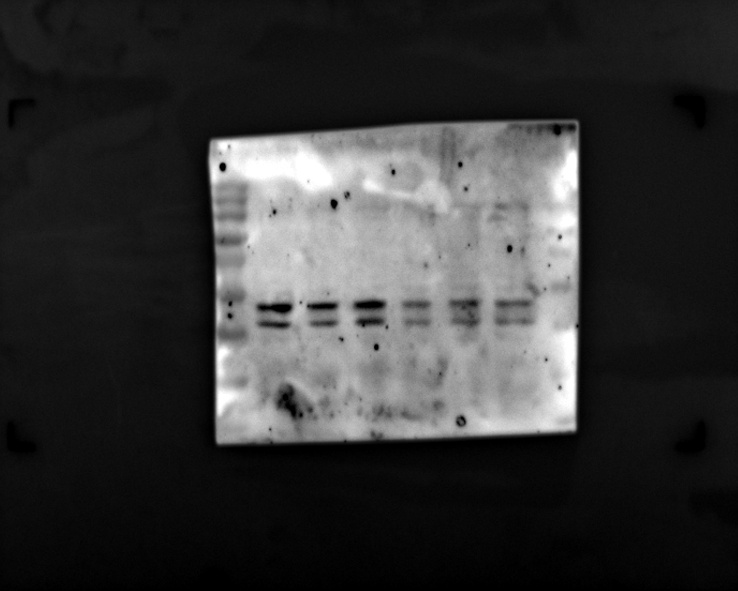


β-actin HSC


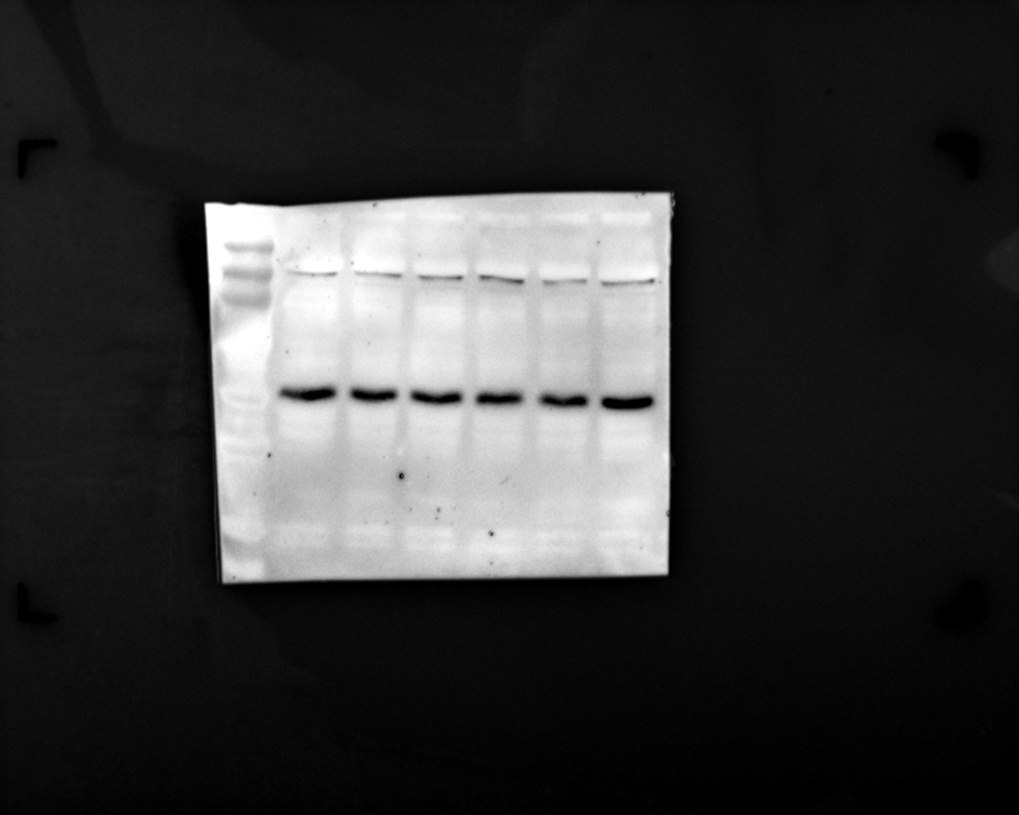


α-SMA JS-1


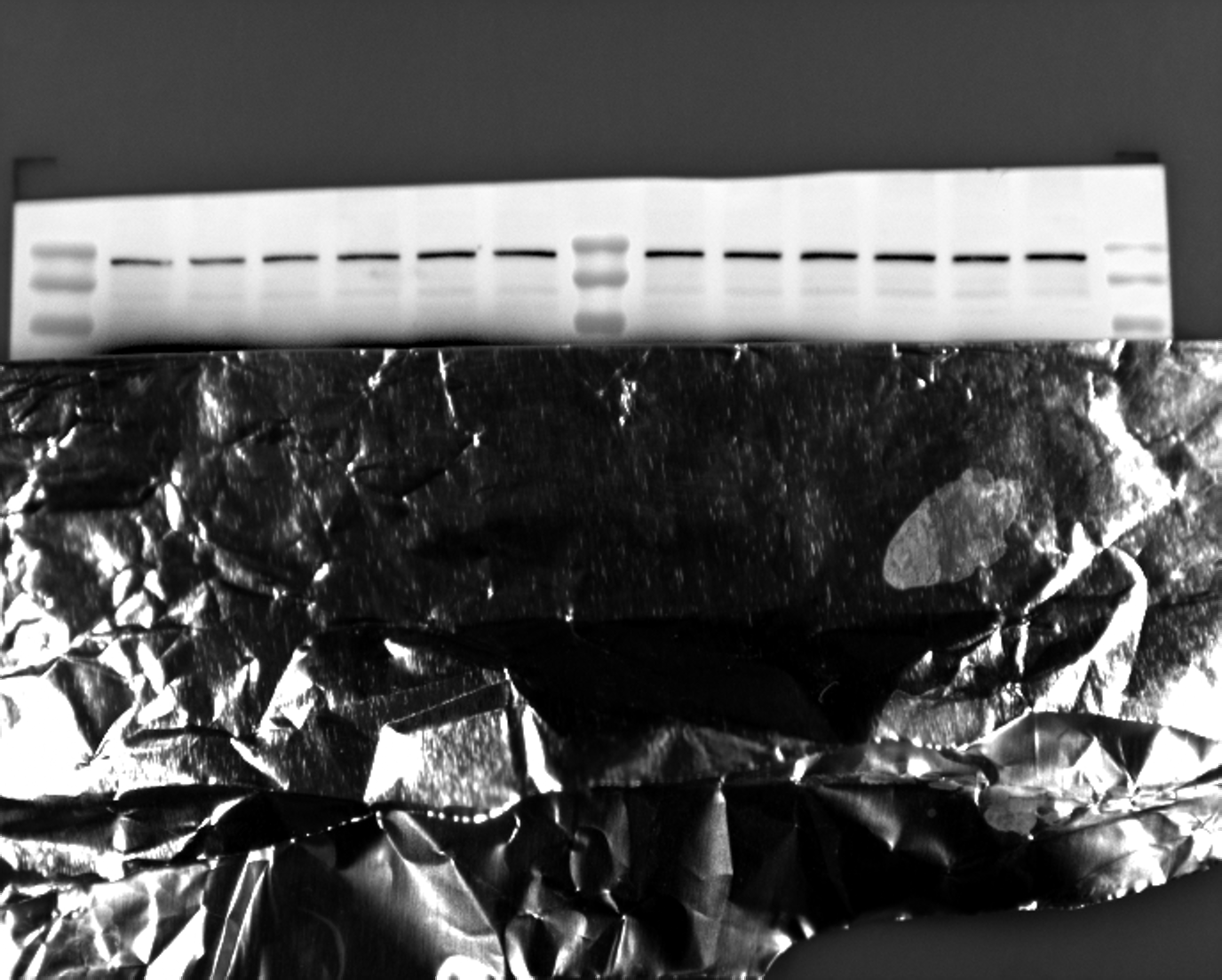


COL1A1 JS-1


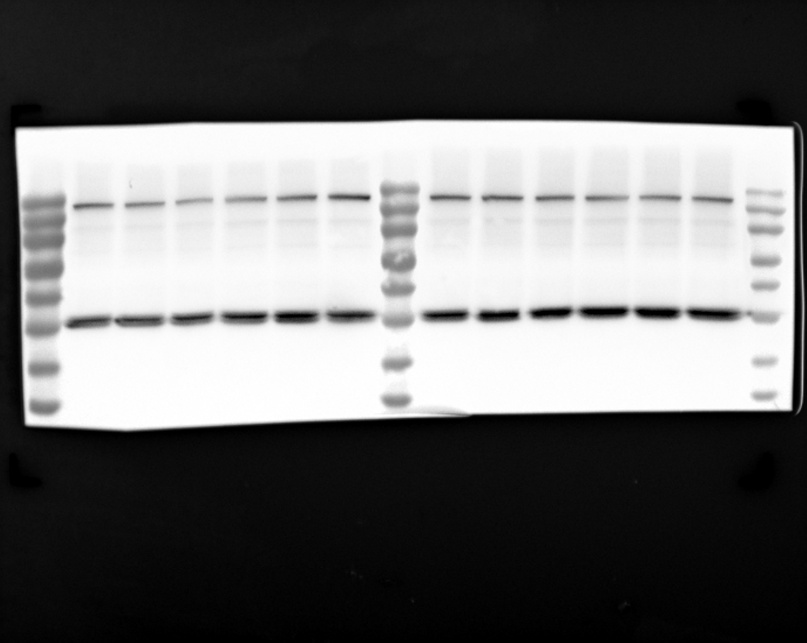


METTL3


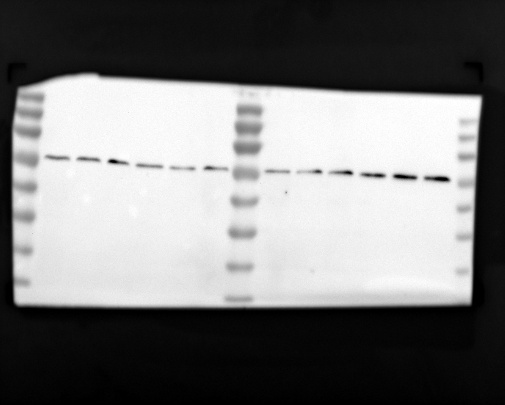


METTL14 JS-1


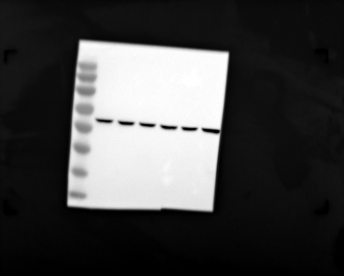


FTO JS-1


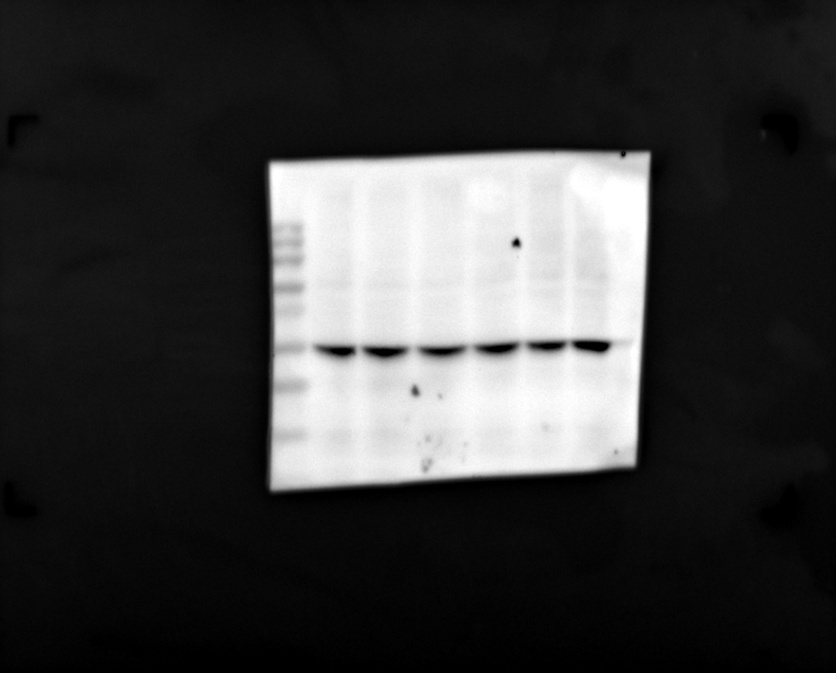


Tubulin-a


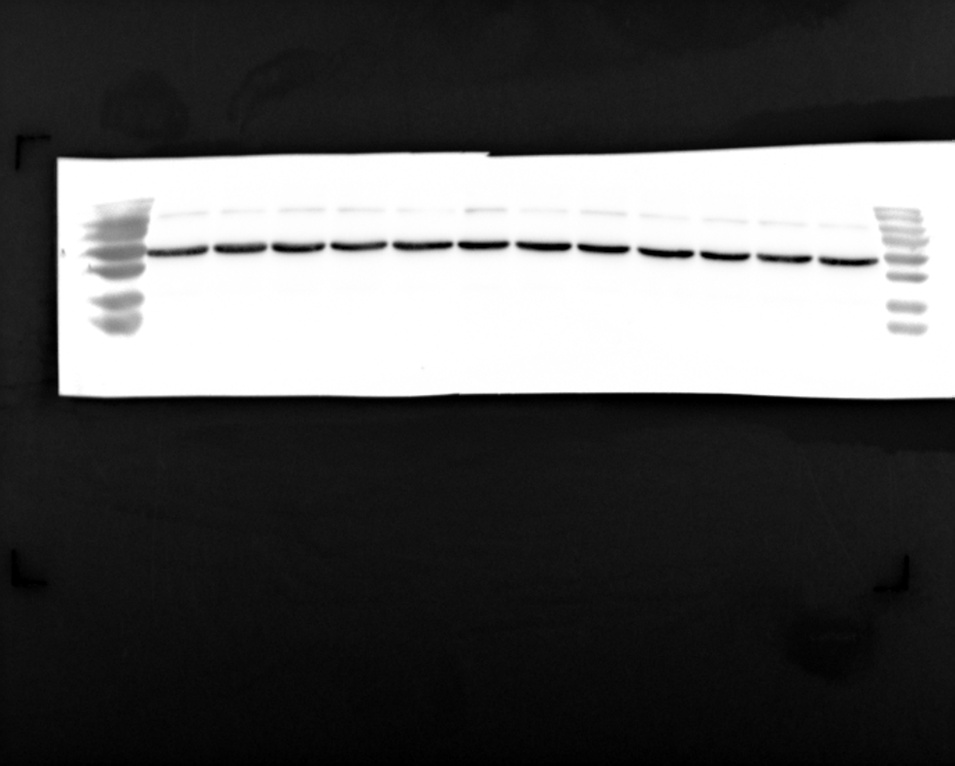


**Fig. 4**

METTL3 SiRNA


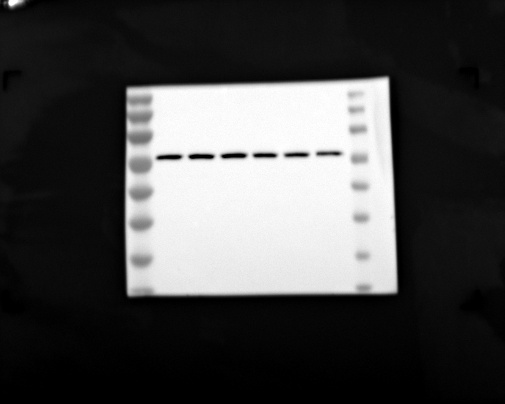


Tubulin-a


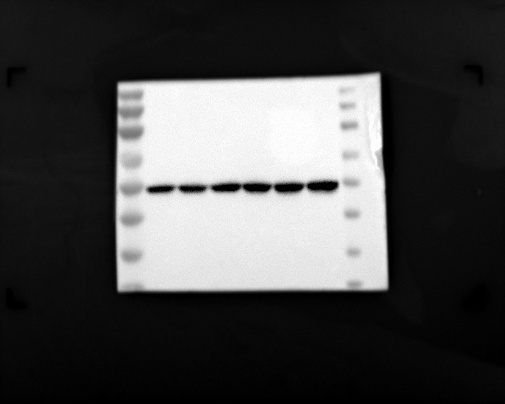


a-SMA


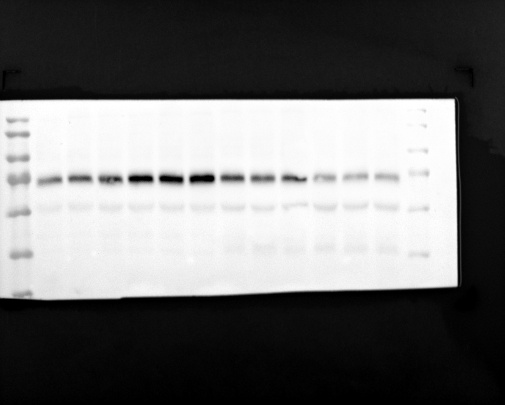


COL1A1


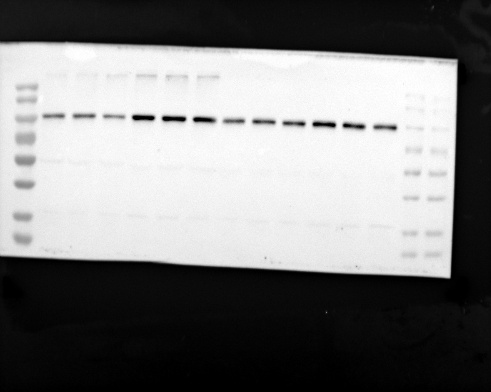


Tubulin-a


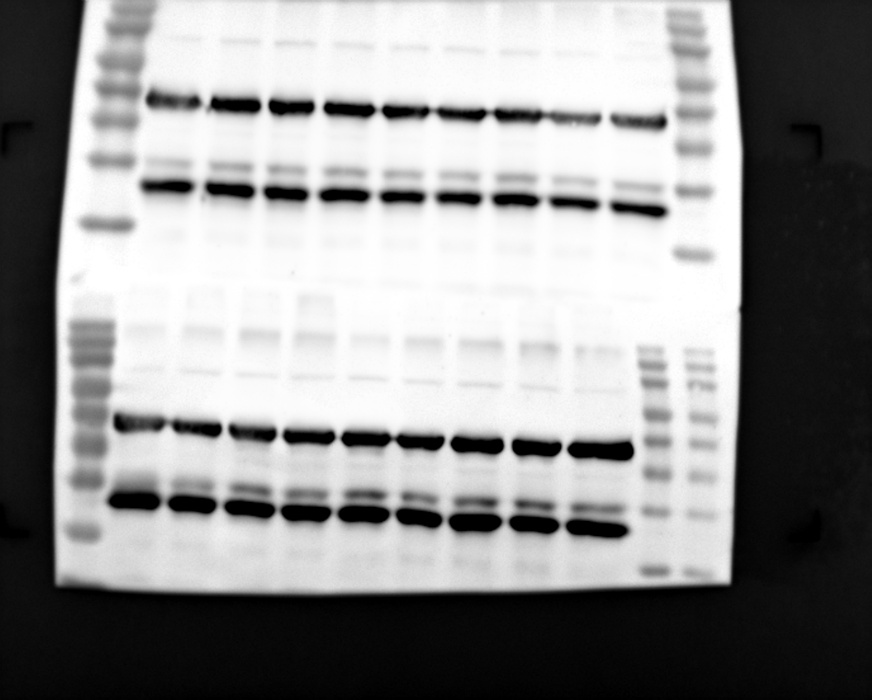


a-SMA


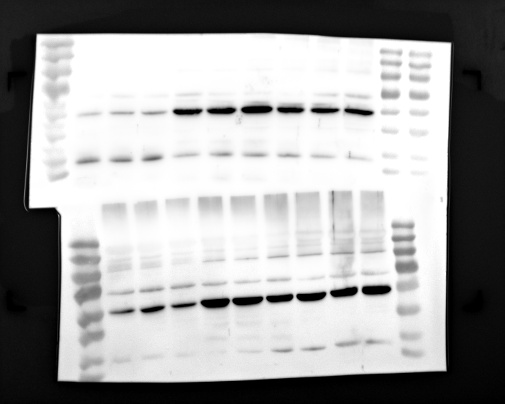


COL1A1


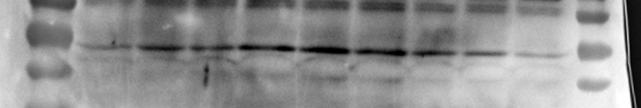


Tubulin-a


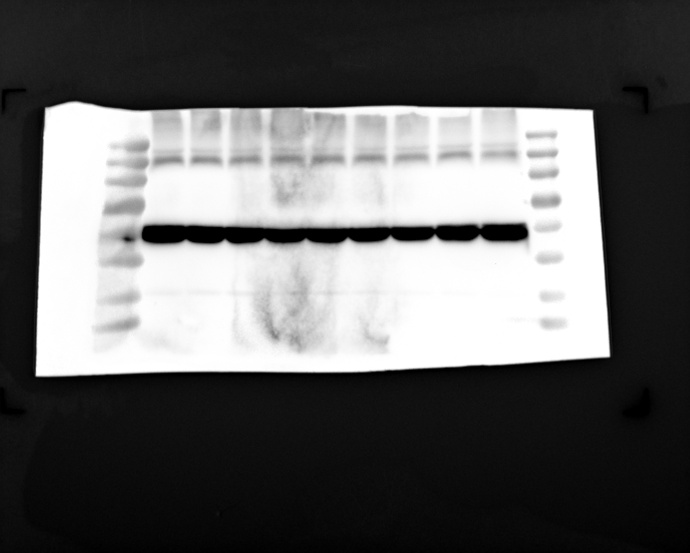


a-SMA


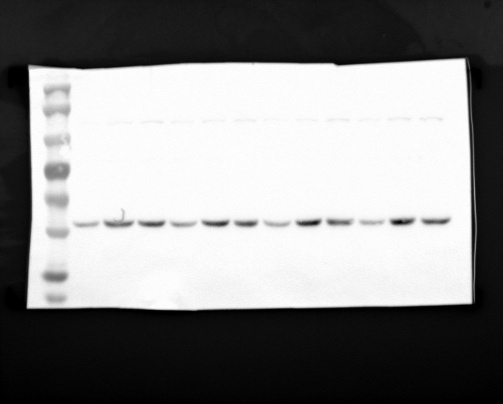


COL1A1





Tubulin-a


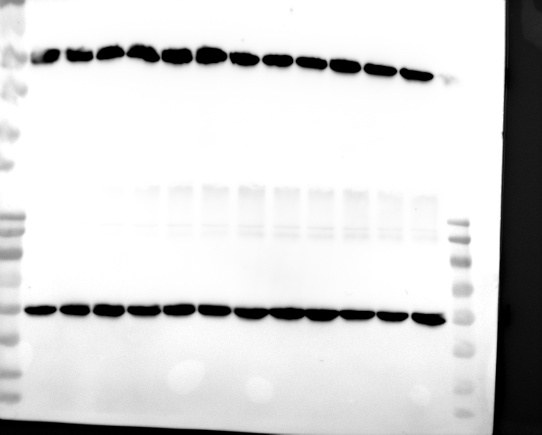


**Fig. 5**

YTHDF1


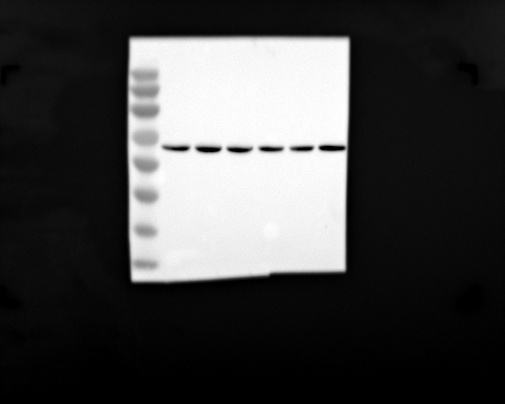


YTHDF2


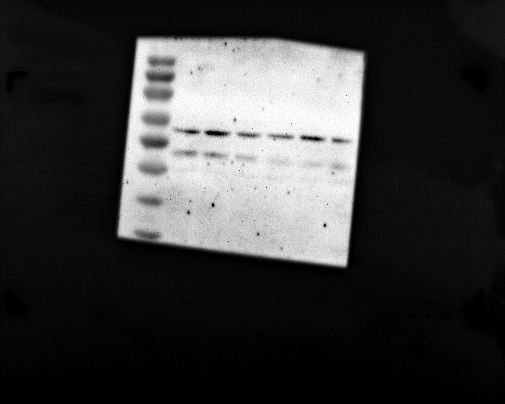


YTHDF3


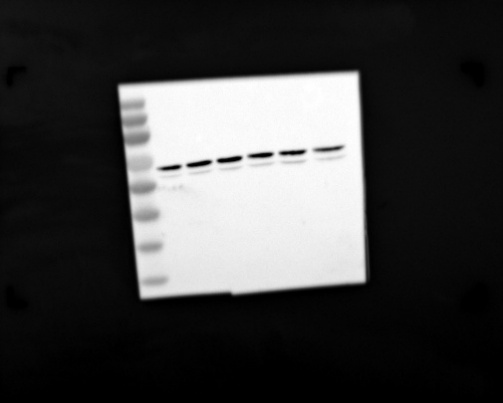


Tubulin-a


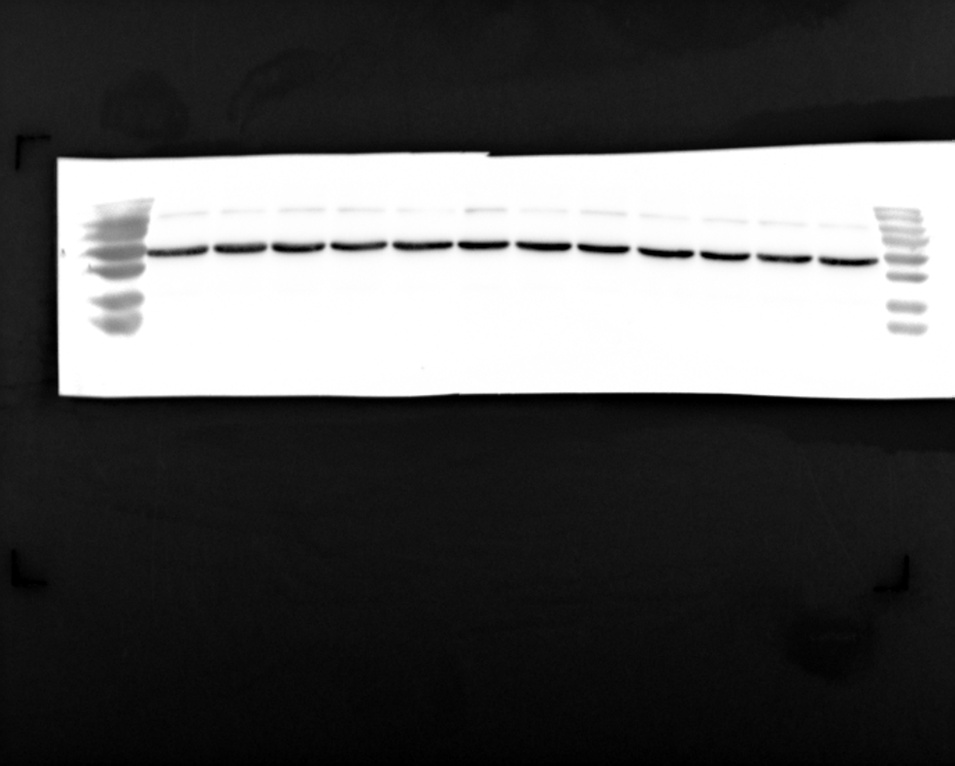


COL1A1 siYTHDF1


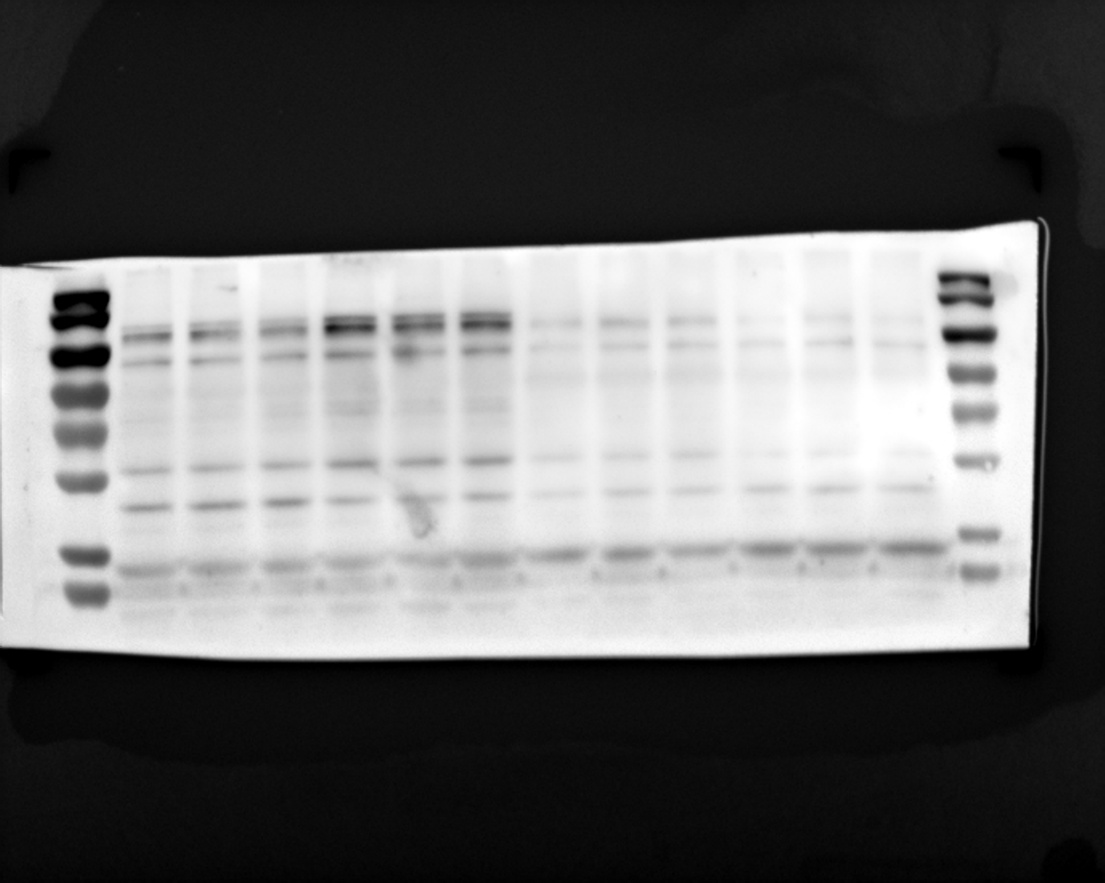


Tubulin-a siYTHDF1


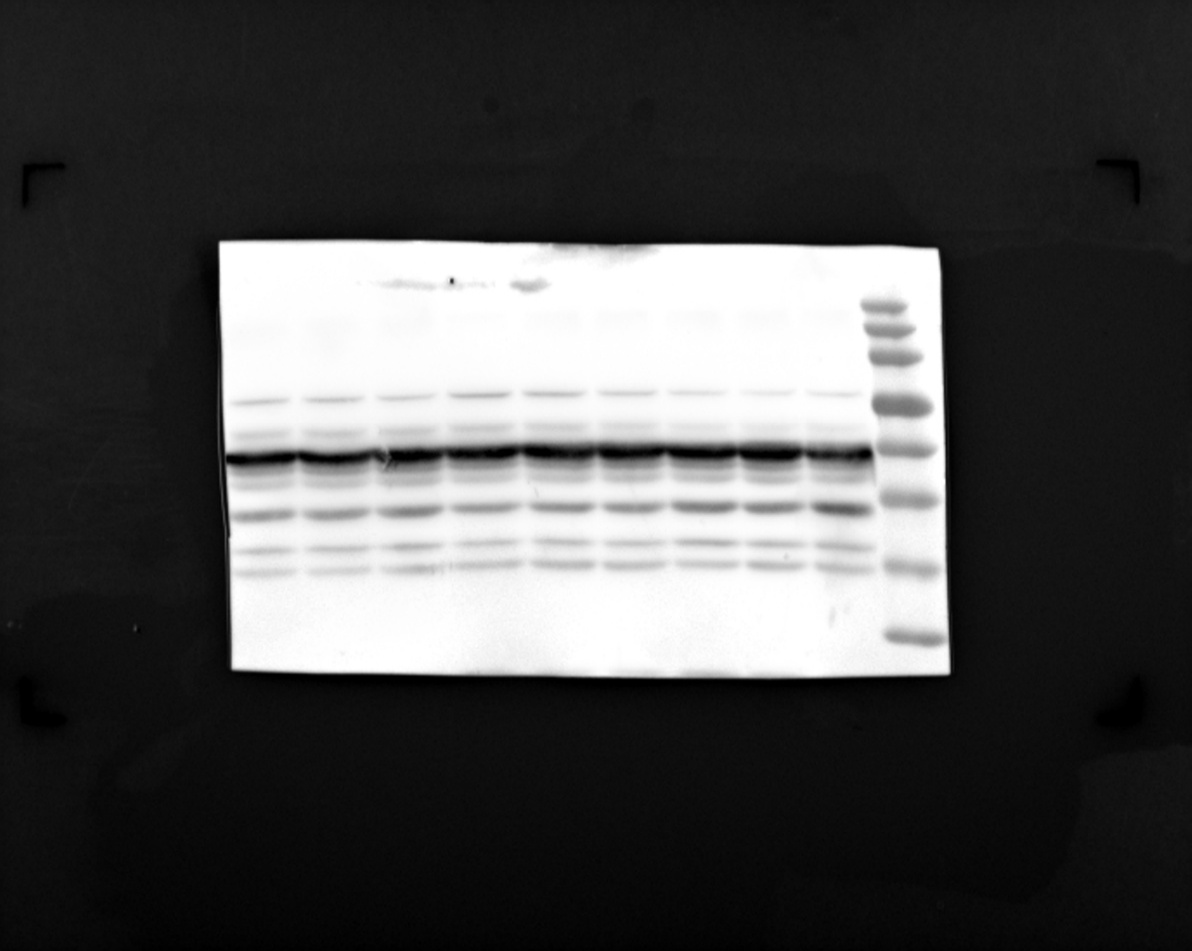


**Fig. 6**

METTL3 SAX





Tubulin-a


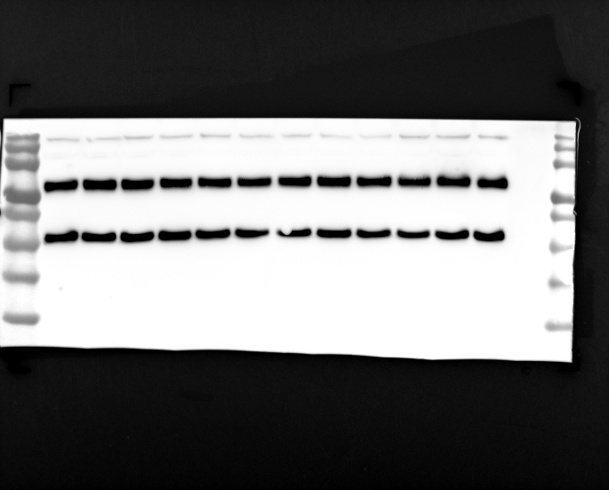


COL1A1 SAX


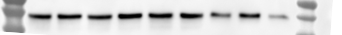


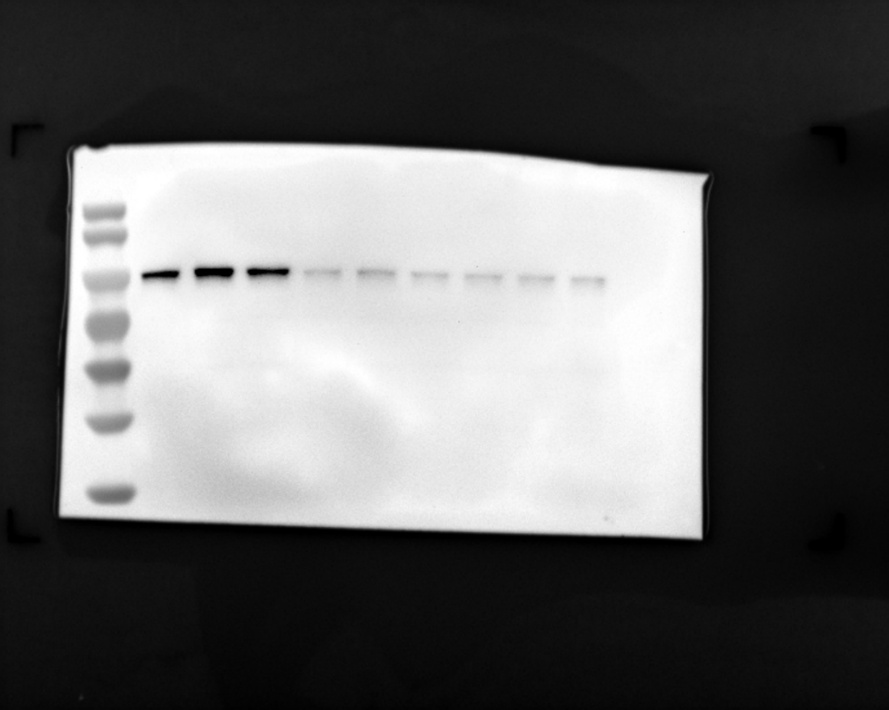
DPP-4

Tubulin-a


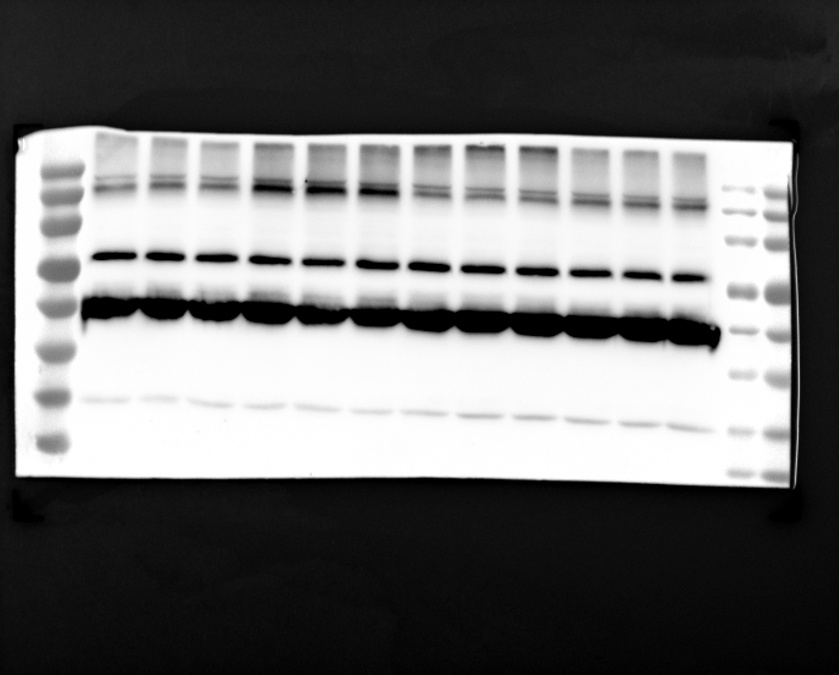


COL1A1





Tubulin-a


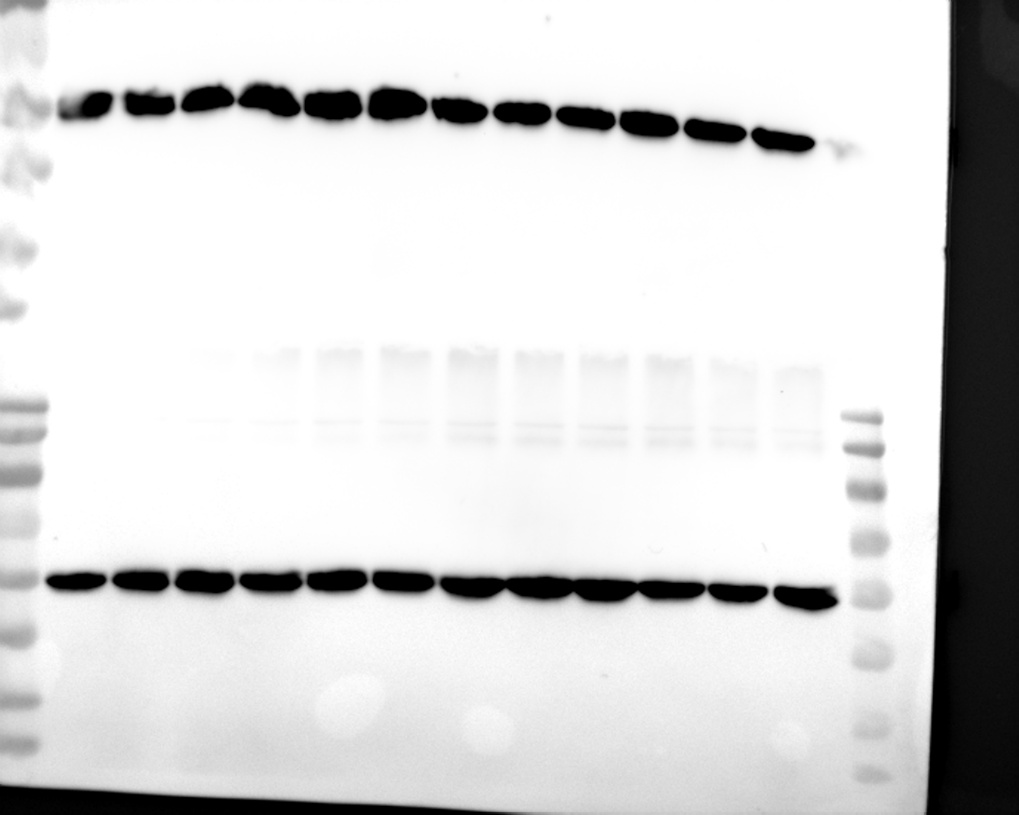

Supplement: Supplementary file 2 — Additional file 2: Original Western blot images. [file 40104_2026_1380_MOESM2_ESM.docx]
